# Estimation of the biaxial tensile behavior of ovine esophageal tissue using artificial neural networks

**DOI:** 10.1186/s12938-024-01296-y

**Published:** 2024-10-12

**Authors:** H. M. Ngwangwa, D. Modungwa, T. Pandelani, F. J. Nemavhola

**Affiliations:** 1 Department of Mechanical, Bioresources and Biomedical Engineering, School of Engineering and the Built Environment, College of Science, Engineering and Technology, University of South Africa, Private Bag X6, Florida, 1710 South Africa; 2 Peace, Safety and Security, Council for Scientific and Industrial Research, PO Box 395, Pretoria, 0001 South Africa; 3 Department of Mechanical Engineering, Faculty of Engineering and the Built Environment, Durban University of Technology, Durban, South Africa

**Keywords:** NARX network, Esophageal tissue, Stress–strain response, Holzapfel model

## Abstract

Diseases of the esophagus affect its function and often lead to replacement of long sections of the organ. Current healing methods involve the use of bioscaffolds processed from other animal models. Although the properties of these animal models are not exactly the same as those of the human esophagus, they nevertheless present a reasonable means of assessing the biomechanical properties of the esophageal tissue. Besides, sheep bear many similarities physiologically to humans and they also suffer from same diseases as humans. The morphology of their esophagus is also comparable to that of humans. Thus, in the study, an ovine esophagus was studied. Studies on the planar biaxial tests of the gross esophageal anatomy are limited. The composite nature of the gross anatomy of the esophagus makes the application of structure-based models such as Holzapfel-type models very difficult. In current studies the tissue is therefore often separated into specific layers with substantial collagen content. The effects of adipose tissue and other non-collagenous tissue often make the mechanical behavior of the esophagus widely diverse and unpredictable using deterministic structure-based models. Thus, it may be very difficult to predict its mechanical behavior. In the study, an NARX neural network was used to predict the stress–strain response of the gross anatomy of the ovine esophagus. The results show that the NARX model was able to achieve a correlation above 99.9% within a fitting error margin of 16%. Therefore, the use of artificial neural networks may provide a more accurate way of predicting the biaxial stress–strain response of the esophageal tissue, and lead to further improvements in the design and development of synthetic replacement materials for esophageal tissue.

## Introduction

The esophagus is usually defined as the long, hollow tube that connects the mouth via the pharynx to the stomach [1, 3]. Thus, its main function is transportation of a food bolus by means of peristaltic waves from the mouth to the stomach [1, 4, 5]. It is 20 to 25 cm long and passes through three anatomical parts of the body, namely the neck, thorax, and abdomen [6]. The esophagus comprises four distinct structural layers: mucosa, submucosa, muscularis externa, and adventitia [4, 6, 7]. The mucosa itself consists of three layers: the innermost layer, along which food passes; the lamina propria, comprising connective tissue composed of collagen type III and elastin, and the muscularis mucosa, which consists of smooth muscle [6]. The main function of the mucosa is to protect the underlying layers from damage [8]. The submucosa has a complex distribution of collagen and elastin fibers. This layer has high circumferential distensibility while maintaining high axial strength [6]. The muscularis externa is the muscular wall of the esophagus, and is made up of circumferential and longitudinal layers of tissue that dominate peristaltic movement during food transportation.

In terms of its physiological functioning, the esophagus is reported to be capable of being stretched up to 50% under internal pressures of between 3 and 5 kPa [6, 9]. The esophageal tissue is therefore highly elastic and exhibits nonlinear behavior. This nonlinear behavior is advantageous for the physiological functioning of the esophagus in that it ensures high compliance in lower tissue stretches while exhibiting high tissue stiffening in larger stretches, thereby preventing tissue over-dilatation [7].

During food transport, the peristaltic waves travel at 3 to 4 cm/s; they last for about 3 to 4.5 s and reach peak pressure of 60 to 140 mmHg [5, 7, 10, 11]. The esophageal tissue contracts longitudinally, thereby increasing the number of fibers lying along the circumferential direction that bear the internal pressure. This results in the reduction in hoop stress that is borne by each fiber in the circumferential direction. It is reported that the amount of stress carried by each fiber is reduced to one-third of its stress in the absence of such contraction [12].

Esophageal cancer accounts for an estimated 400,000 deaths annually, and it is reported to be the sixth leading cause of cancer-related deaths worldwide [6, 13]. Most esophageal cancer treatment methods necessitates dissection of long sections of the esophagus [6, 14, 15]. The same is true for other forms of esophageal pathologies due to the inability of the tissue to regenerate [2, 16]. Thus, there is always a need in such cases to find suitable bioscaffolds to preserve functionality of the esophagus. However, this is where the biggest challenge lies: surgical treatments of the esophagus will not always result in complete functional recovery, often leading to decreased quality of life and survival rate [14, 17]. There remains a lack of suitable replacement materials with similar esophageal mechanical properties [17]. Owing to its inherent structural complexity, it is difficult to manufacture such tissue such that it perfectly replicates the mechanical behavior of the natural esophagus. The esophagus is a composite muscle whose different layers possess different mechanical properties with variations along its span in different anatomical zones. Thus, in order to produce a biomaterial for the replacement of this tissue, an understanding of its mechanical properties is necessary. The use of accurate mathematical models is an inexpensive and ethically acceptable way of achieving such a purpose.

In the study, the widely published Holzapfel model [18–20] was investigated in modeling ovine esophageal tissue. The Holzapfel model, with its structural-modeling basis, is reported to be one of the most suitable models for modeling multi-layered biological tissue such as the esophagus, as it was originally formulated for the modeling of arterial walls [20]. However, there is a complexity underlying the modeling of esophagus tissue in that its mechanical behavior varies widely in different anatomic zones and becomes extremely difficult to predict accurately in the presence of minor variations in tissue location and operating conditions [5, 21, 22]. Therefore the study investigated the capability of an artificial neural network (ANN) of the nonlinear autoregressive with exogenous input (NARX) type [23]. Despite the esophageal tissue having a characteristic hyperelastic nonlinear behavior, it is highly random and the stretch–stress curves differ widely from one test to another, such that prediction of mechanical behavior becomes extremely difficult. Thus, the use of the ANN-based approach is deemed a viable approach to the modeling of this type of tissue.

## Theoretical framework

### Holzapfel model

Holzapfel [24, 25] proposed a structurally based model whose strain energy function (SEF) is given by:

1 $$W={W}_{iso}+{W}_{aniso},$$

where ${W}_{iso}$ denotes the isotropic component of the SEF and ${W}_{aniso}$ represents the anisotropic part of the SEF. The isotropic contribution is associated with the mechanical response of the non-collagenous components of the tissue and is given by ${W}_{iso}={c}_{10}\left({I}_{1}-3\right)$[18, 24, 26]:

2 $$W=\frac{c}{2}\left({I}_{1}-3\right)+\frac{{k}_{1}}{{k}_{2}}{e}^{\left[{k}_{2}{\left({I}_{4}-1\right)}^{2}\right]}-1,$$

where ${I}_{1}={\lambda }_{\theta }^{2}+{\lambda }_{z}^{2}+{\lambda }_{r}^{2}$ is the first invariant representing material dilatation or volume change in the material; ${I}_{4}={\lambda }_{\theta }^{2}{\text{cos}}^{2}\theta +{\lambda }_{z}^{2}{\text{sin}}^{2}\theta ;$ $c$ and ${k}_{1}$ are stress-like parameters; ${k}_{2}$ is a dimensionless parameter; and $\theta$ is the angle between the collagen fiber orientation and the circumferential direction.

Cauchy stresses can be expressed as derivatives of the SEF with regard to strain measures thus [22, 25, 26]:

3 $${\sigma }_{aa}^{W}={\lambda }_{a}\frac{\partial W}{\partial {\lambda }_{a}}-p\,\,\,\, a=\theta , z, r,$$

where $p$ represents the hydrostatic pressure and can be calculated from boundary conditions. Assuming negligible shear stresses during tissue testing, the Cauchy stresses can then be derived as [26]:

4 $${\sigma }_{\theta \theta }^{W}=2\left({\lambda }_{\theta }^{2}-{\lambda }_{\theta }^{-2}{\lambda }_{z}^{-2}\right)\frac{\partial W}{\partial {I}_{1}}+2{\lambda }_{\theta }^{2}{{cos}}^{2}\theta \frac{\partial W}{\partial {I}_{4}},$$

5 $${\sigma }_{zz}^{W}=2\left({\lambda }_{z}^{2}-{\lambda }_{z}^{-2}{\lambda }_{\theta }^{-2}\right)\frac{\partial W}{\partial {I}_{1}}+2{\lambda }_{\theta }^{2}{{sin}}^{2}\theta \frac{\partial W}{\partial {I}_{4}}.$$

In the solution of the Cauchy stresses, the optimal material parameters $c, {k}_{1}, {k}_{2} and \theta$ are determined through an optimization process. The global minimum of the following objective function is sought [22, 26]:

6 $$\epsilon = \sum\limits_{{i = 1}}^{n} {\left[ {\left( {\sigma _{{\theta \theta }} - \sigma _{{\theta \theta }}^{W} } \right)_{i}^{2} + \left( {\sigma _{{zz}} - \sigma _{{22}}^{W} } \right)_{i}^{2} } \right]} \,n{\mkern 1mu} {\mkern 1mu} data{\mkern 1mu} {\mkern 1mu} points.$$

The Pearson correlation coefficient (${R}^{2}$) and error measure $\epsilon$ based on ${\epsilon }^{2}$ are calculated for each direction as follows:

7 $$\frac{1}{{\sigma }_{ref}}\sqrt{\frac{{\epsilon }^{2}}{n-q},}$$

where $n$ is the total number of data points in the sequence, $q$ is the number of parameters, and ${\sigma }_{ref}=\frac{\sum {\sigma }_{aa}^{W}}{n}$.

### NARX model

**Fig. 1:**
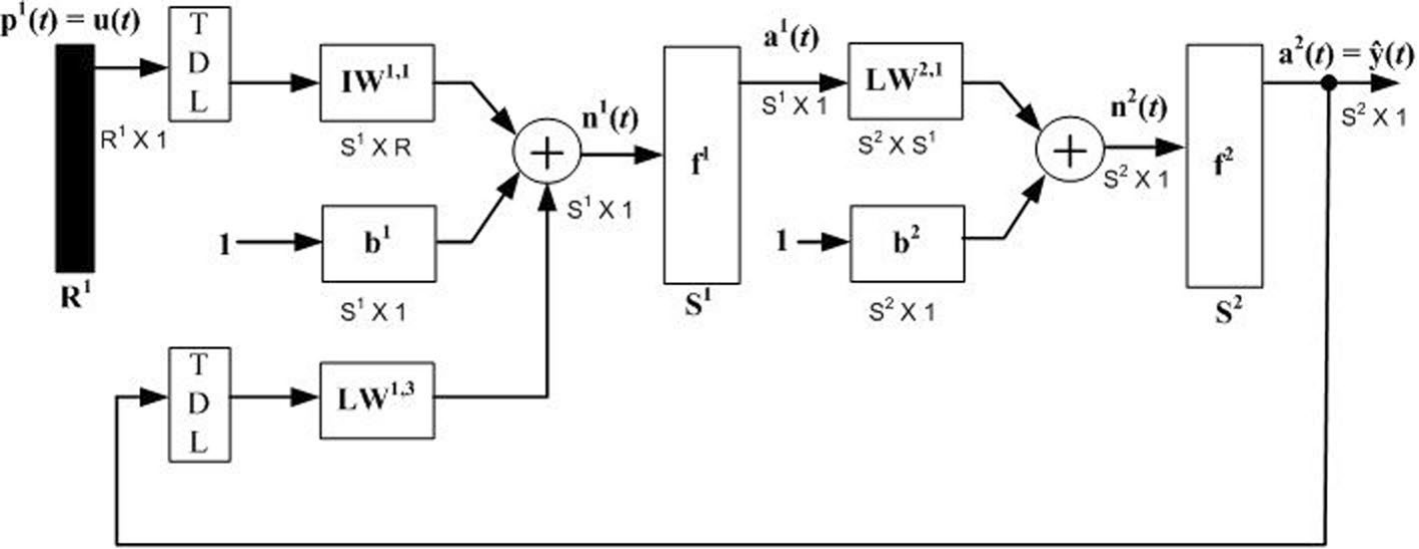
The architecture of the NARX network. The network is 1-5-1 with one input, five hidden neurons and one pure linear output layer. The training data are divided through a random generator and training is performed by a Bayesian regularization which ensures that an optimal number of iterations are executed

An NARX model with 1-5-1 architecture as shown in Fig. 1 was employed in the study. The model is a nonlinear autoregression with exogenous inputs (NARX) [27, 28]. This implies that the network has feedback delays as part of its inputs; for this particular network, two feedback delays were found to be sufficient.

More delays simply resulted in longer computational periods and overfitting of the target. As shown in Fig. 1, the network calculates its output $\widehat{y}\left(t\right)$ by means of the following expression:

8 $$\widehat{y}\left(t\right)={f}^{2}\left\{{f}^{1}\left[{\mathbf{I}\mathbf{W}}^{\text{1,1}}\cdot u\left(t\right)+{\mathbf{b}}^{1}+{\mathbf{L}\mathbf{W}}^{\text{1,3}}\cdot \widehat{y}\left(t\right)\right]\cdot {\mathbf{L}\mathbf{W}}^{\text{2,1}}+{\mathbf{b}}^{2}\right\}.$$

The function ${f}^{1}\left(.\right)$ is a nonlinear “tansigmoid” mapping function which is implemented in the neural network by the autoregression expression given by [23, 27–31]:

9 $${f}^{1}\left(.\right)=tansig\left({{\varvec{I}}{\varvec{W}}}^{\text{1,1}}\cdot u\left(t\right)+{{\varvec{b}}}^{1}+{{\varvec{L}}{\varvec{W}}}^{\text{1,3}}\cdot \widehat{y}\left(t-n\right)+g\left(\epsilon \left(t\right)\right)\right),$$

where $u\left(t\right)$ is the strain at time t, $\widehat{y}\left(t-n\right)$ is the network predicted stress value at time $t-n$ and $\varepsilon \left(t\right)$ is the error function given by the white noise. In the study, only two time delays were applied. The application of $\varepsilon \left(t\right)$ helps in improving the generalization of the network. $\mathbf{I}\mathbf{W}$ and $\mathbf{L}\mathbf{W}$ are matrices that contain adjustable values, and are technically referred to as input layer and hidden layer matrices. The term **b** denotes a vector containing network bias**.** The main neural network function therefore is to adjust the values in the weight matrices and bias vector in order to minimize the error between its target and network calculated output. The output layer function ${f}^{1}\left(.\right)$ is a pure linear function.

The process of adjusting the network weight and bias parameters is implemented in the NARX network through a backpropagation algorithm. The backpropagation algorithm works by ploughing back the errors calculated at the output layer through the weights and biases in the hidden and input layers. New weight connecting nodes A and B after the (j + 1)th training epoch are calculated as:

10 $${W}_{j+1}^{AB}={W}_{j}^{AB}+\left({\varepsilon }_{j}^{B}{\widehat{y}}_{j}^{A}\right),$$

where $W$ in this case represents a weight element within the weight matrices connecting two nodes, ${\varepsilon }_{j}^{B}$ is the error calculated after the jth epoch at node B, and ${\widehat{y}}_{j}^{A}$ is the output calculated at node A after the jth epoch. The error ${\varepsilon }_{j}^{B}$ can be calculated by:

11 $${\varepsilon }_{j}^{B}={\widehat{y}}_{j}^{B}\left(1-{\widehat{y}}_{j}^{B}\right)\left(\sum_{i=1}^{N}{W}_{j}^{B{X}_{i}}{\varepsilon }_{j}^{{X}_{i}}\right),$$

where i represents a node number within a particular layer; thus the superscript ${X}_{i}$ simply denotes the ith node in an Xth layer. This representation has been used here for mathematical convenience. The term ${\widehat{y}}_{j}^{B}\left(1-{\widehat{y}}_{j}^{B}\right)$ is included because of the utilization of the tansigmoid function, and is a result of taking the partial derivative of this function with respect to the weights.

### Data preparation

Because the training was a dynamic process, the data were converted into sequences. This implies that the outputs were not expected to remain stationary with time, and the network outputs were delayed by some duration from the inputs. The data were further normalized based on the maximum values of stress in the tests to avoid saturation. As shown later in this study, network saturation can induce very large errors during network simulation. The training data were divided into 50%, 25%, and 25% representing training, validation, and testing proportions, respectively. The data division was carried out randomly along the data sequence. This implies that the data division algorithm sampled out the numbers to be allocated for training, validation and testing differently for each iteration. Such random sampling at each iteration ensured that the network was able to learn as many features from the training data as possible.

### Network regularization

During network training the sum of squared errors is typically minimized; however, in this study the Bayesian regularization was employed. The implementation of regularization ensured that not only the sum of square errors, but also the sum of squares of the network weights was minimized. This process ensured that the network did not overlearn the training data, which could have resulted in fitting the unessential parts of the training data, thereby failing to accurately simulate new test data. In this way, the objective function can be written as [32]:

12 $$F\left({\varvec{w}}\right)=\beta {E}_{D}+\alpha {E}_{{\varvec{w}}},$$

where $\mathbf{w}$ represents the vector of weights, ${E}_{D}$ denotes the sum of squared errors, ${E}_{w}$ is the sum of squared errors of the network weights, and $\alpha$ and $\beta$ are the parameters of the objective function that are to be optimized. In the Bayesian regularization process, the network weights are initialized as random variables; then, after the data are applied, the density function of the weights is updated according to the Bayes rule [32, 33]:

13 $$P\left({\varvec{w}}|D,\alpha ,\beta ,M\right)=\frac{P\left(D|{\varvec{w}},\beta ,M\right)P\left({\varvec{w}}|\alpha ,M\right)}{P\left(D|\alpha ,\beta ,M\right)},$$

where the term $P\left(x|y\right)$ is taken to imply the conditional probability of an event x given that an event y has occurred; D represents the stress data set, M denotes the NARX model, and the other parameters are as defined in Eq. (12).

**Fig. 2:**
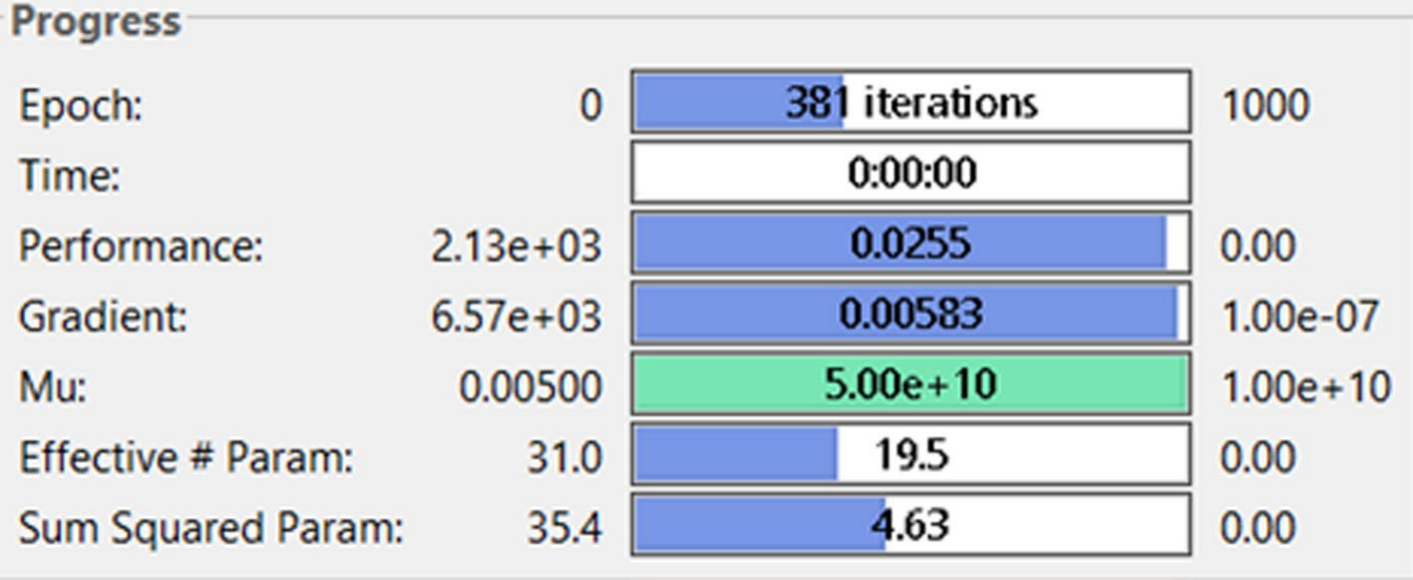
Typical example of the regularization training. The network usually stops the training process after certain goals have been met without necessarily attempting to complete all the default training values

The network was trained cyclically five times with noise at different magnitudes to improve its generalization [34]. As shown in the network performance graphic in Fig. 2, the ANN stopped training at slightly below 400 out of a possible 1000 iterations with a mean squared error (mse) = 0.026 and gradient of 0.00583. The network converged after reducing the number of training parameters and sum of squared parameters to just 19.5 effective training parameters and 4.63 sum of squared parameters from a total of 31 training parameters and 35.4 squared parameters, respectively. It is clear that this reduction in both the total number of training parameters and sum of squared parameters resulted in the reduction of computational overload during testing or ANN simulation [32].

### Identification of training data

**Fig. 3:**
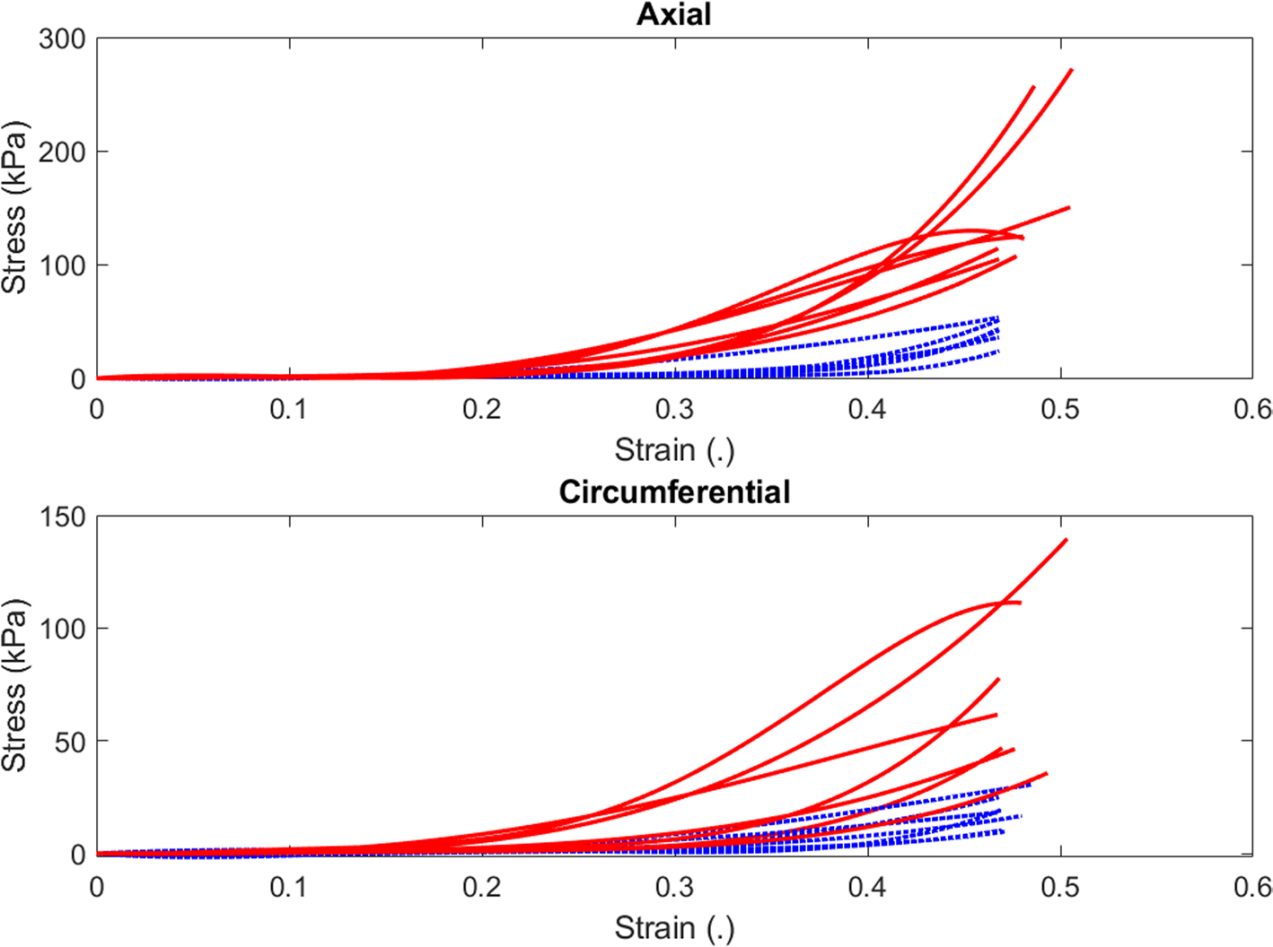
Two broad categories of the training data for both axial and circumferential stress–strain responses. The data were classified into ‘Good training data’ (solid red) and ‘Poor training data’ (dotted blue). Good training data: Test 2, Test 6, Test 8, Test 10, Test 11, Test 12, and Test 14 (the average data). Poor training data: Test 1, Test 3, Test 4, Test 5, Test 7, Test 9, and Test 13

Initially, a number of iterative trial-and-error steps were conducted to identify the most suitable artificial neural network to be employed for the solution of this problem. A 1-5-1 NARX network was found to yield promising results within a reasonable length of training duration. Only two output delays were found to be satisfactory. However, the identification of training data with enough characteristic features was found to be critical to the accuracy of the neural network. As a first step, the statistical average stress–strain data were calculated from all the 13 tests. The understanding was that such average stress–strain response bears the highest cross-correlation with all the other tests and would reveal important features to be extracted for network training. It was also equally important to examine the character of the training data that yielded the poorest results. Thus, all the other data were applied to the neural network. These stress data were plotted in one graph in both axial and circumferential directions so as to observe their different characters. Two broad categories emerged, as depicted in Fig. 3, where solid lines represent the ‘Good training data’ and dotted lines represent the ‘Poor training data’. It is interesting to note that all except two of the tests fell into only one class for both axial and circumferential directions.

Some of the ‘Poor training data’ tests actually yielded fitting errors in excess of 200% at correlation levels of below 70%. On the other hand, the same neural network would yield fitting errors below 20% at correlation levels of greater than 99%.

**Fig. 4:**
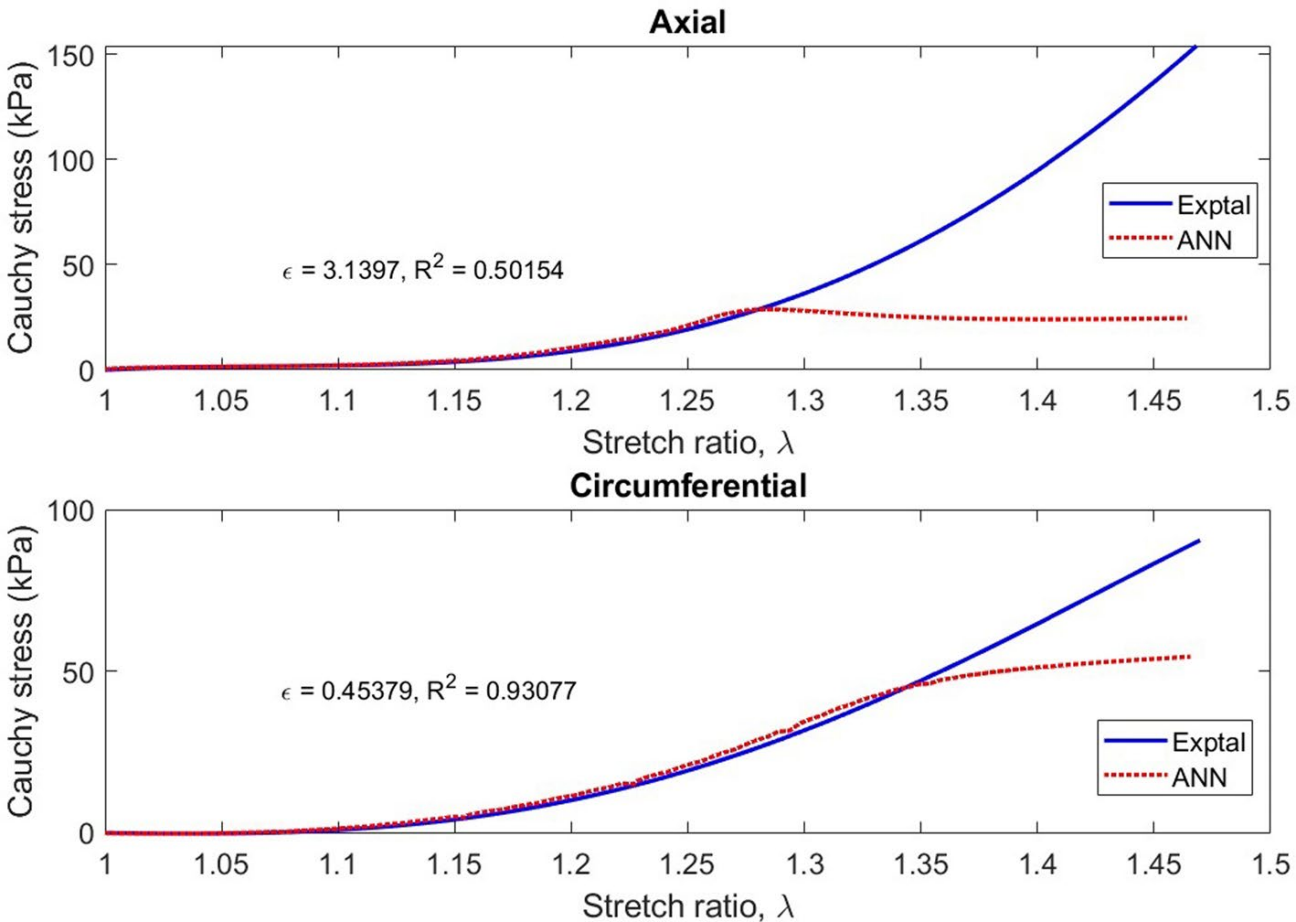
Typical results obtained when ANN estimated stresses were plotted over the experimental data. All raw experimental data, irrespective of test number, typically yielded similar results as shown in the graph where the NN estimates peaked and flattened out slightly below the Cauchy stress of 50 kPa. These errors were resolved by normalization of all training and test data

Two important features were identified from the ‘Good training data’ as opposed to the ‘Poor training data’. These features were the slope and maximum values of the stress data. Poor training data had very small slopes and maximum stress levels such that when the raw data were used to train the network, they yielded errors due to saturation. The low slope retarded the training process to a point where the network often reached maximum epochs without necessarily attaining any convergence. The ‘Poor training data’ curves were close to the horizontal stretch axis over a very long stretch up to a ratio of 1.4. These features revealed that it was possible to reduce the training data to a mere linear curve with sufficient slope to approximate the tangent modulus of the esophageal tissue. The following linear curve was thus fitted to the ‘Good training data’:

14 $$y={p}_{1}\times \lambda +{p}_{2},$$

where ${p}_{1}$ is the slope, $\lambda$ is the stretch ratio, and ${p}_{2}$ is the intercept of the line which represents the bias in this test. During training $y$ was used as the target, while strain $\epsilon$ was used as the input data. However, as per this particular neural network some delayed outputs were also fed back into the network to produce the network outputs. In order to treat saturation errors, all data were normalized according to the maximum stress level in the overall test data. Without such normalization, all tests typically yielded the results as shown in Fig. 4. The performance of the ANN was affected by the saturation problems and failed to produce accurate results at larger stretch ratios in which NN simulations flattened out below the Cauchy stress of 50 kPa.

**Fig. 5:**
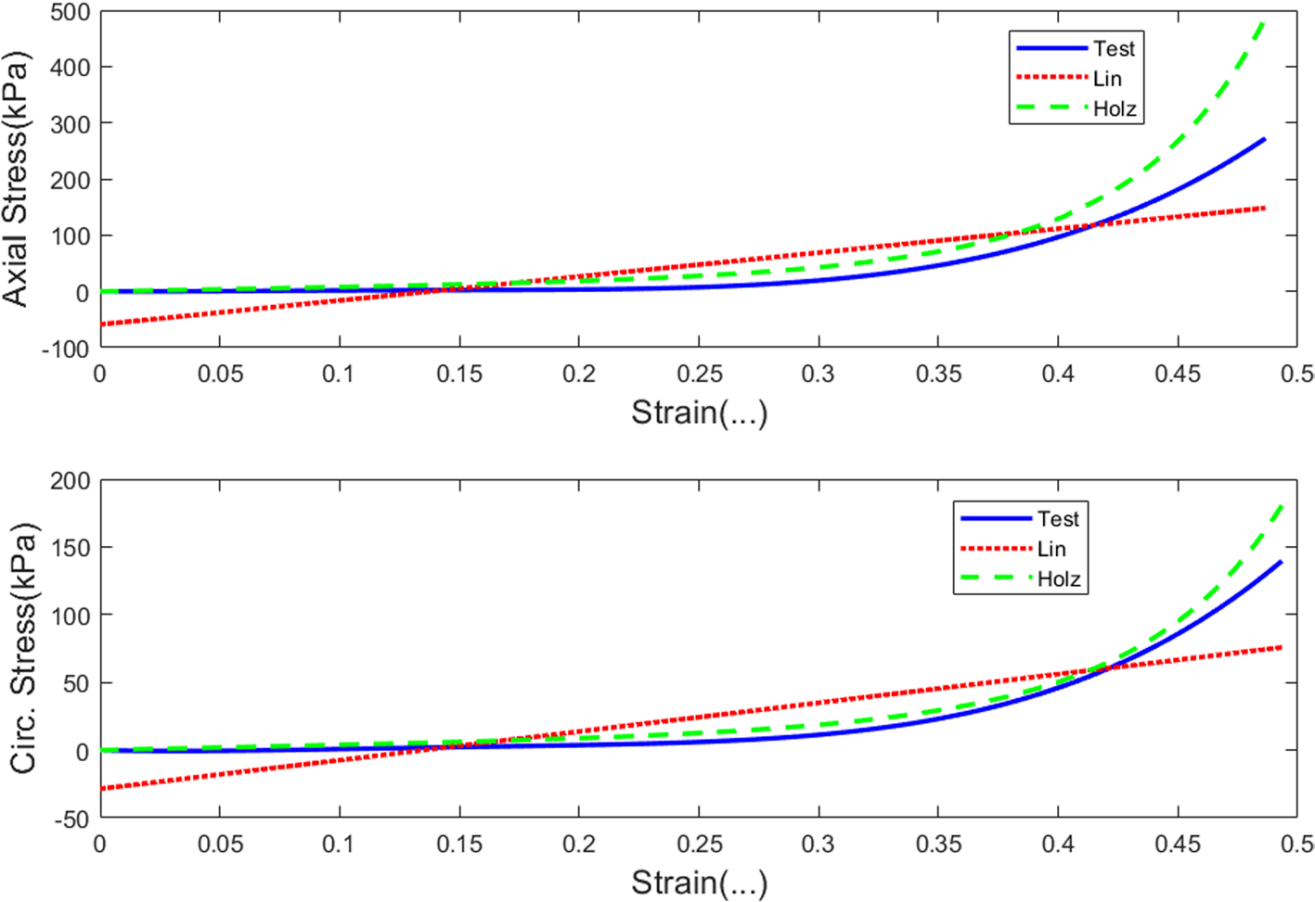
The three types of curves that represent the training data employed in the study. The test data were only used as the control for the study. The blue solid curve labeled as “Test” here represents Test 6, the linear curve is shown as red and dotted (Lin), while the Holzapfel-type curve is presented as a green and dashed line (Holz)

The linear curve can, however, only reveal information about the ultimate tensile stress (as given by the maximum stress level) and the average tissue stiffness, assuming that the nonlinear tissue behavior is linearized. In more physically meaningful terms the linear curve can also be approximated by fitting the classical neo-Hookean model [18, 35] to the test data. However, such a model yields only limited physical interpretation. A more physically insightful approximation of the training data could be constructed by initially fitting the training data with a Holzapfel-type model [18, 20]. It is expected that the use of this model can assist in understanding not only the tissue stiffness, but also tissue stiffening constants, as well as fiber orientation. The linear curve, Holzapfel-type curve, and the raw test data, specifically Test 6 that were investigated in this study as training data are plotted in Fig. 5. In Sect. Results of NARX training performance, the results of the neural network simulations for all these three types of training data are presented.

## Results

### Stress response

Although esophageal tissue comprises characteristically different muscle groups [1, 3], the tensile behavior of esophageal tissue has been observed to resemble that of connective tissue such as tendons, with well-defined toe region, followed by the linear region before the plastic failure region [22, 36–39, 55]. This is due to the fact that biological soft tissue is typically composed of collagen, elastin and proteoglycans. Although they may be differently configured and constituted in different organs, they exhibit similar tensile behavior, with minor variations. For example, esophageal tissue is said to be composed of 20.25% and 18.17% type I collagen in the mucosa–submucosa layer along the longitudinal and circumferential directions [40], respectively, while tendons are composed of 18–24% of type I collagen [41]. Most of these minor differences may be due to adaptation to their different operating conditions and functionalities.

**Fig. 6:**
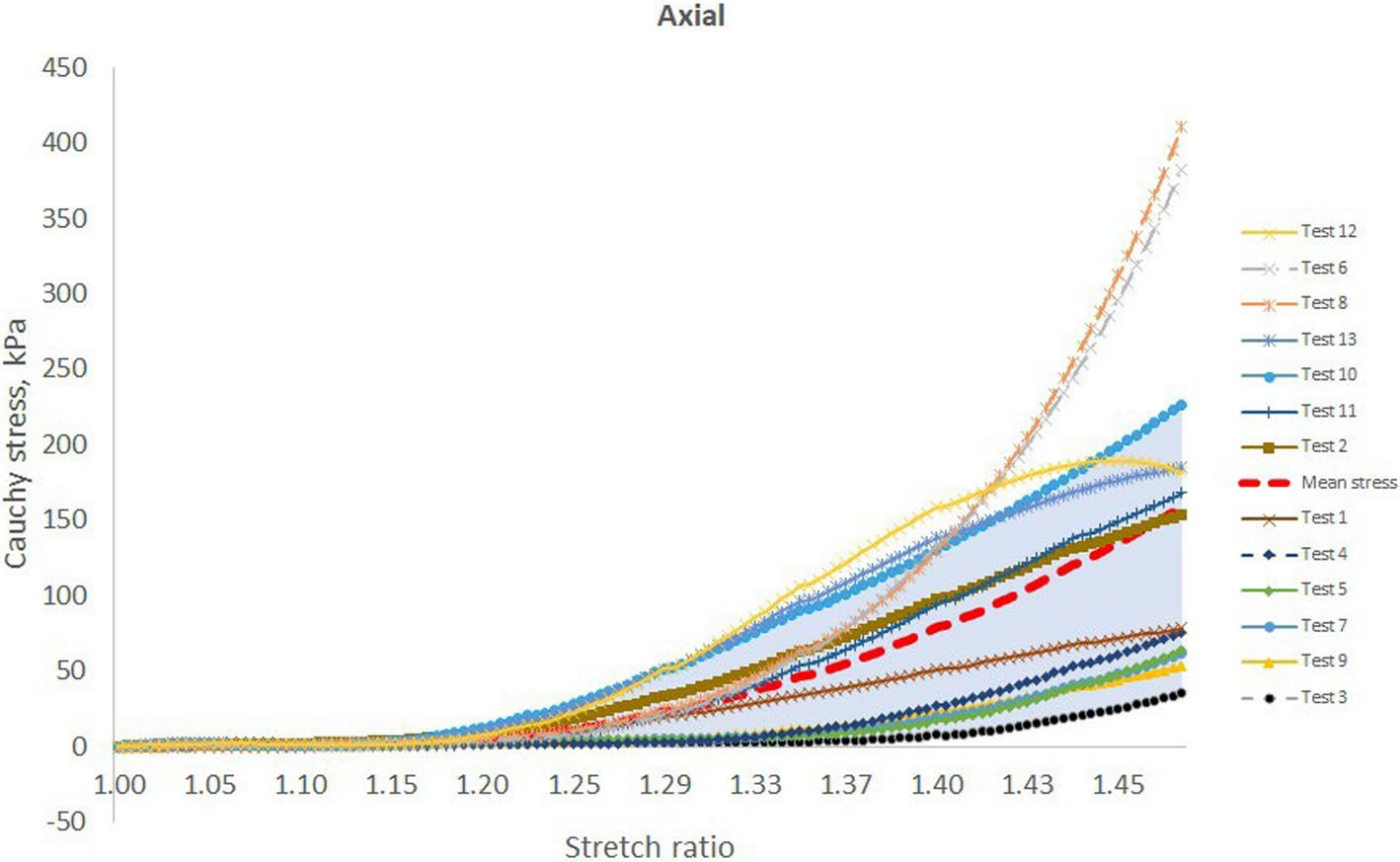
The stretch vs stress curves for the 13 different tests in the study along the axial direction. The mean curve is also included. Note the wide diversion of the curves away from the mean graph, especially in the regions of high tissue stretches

**Fig. 7:**
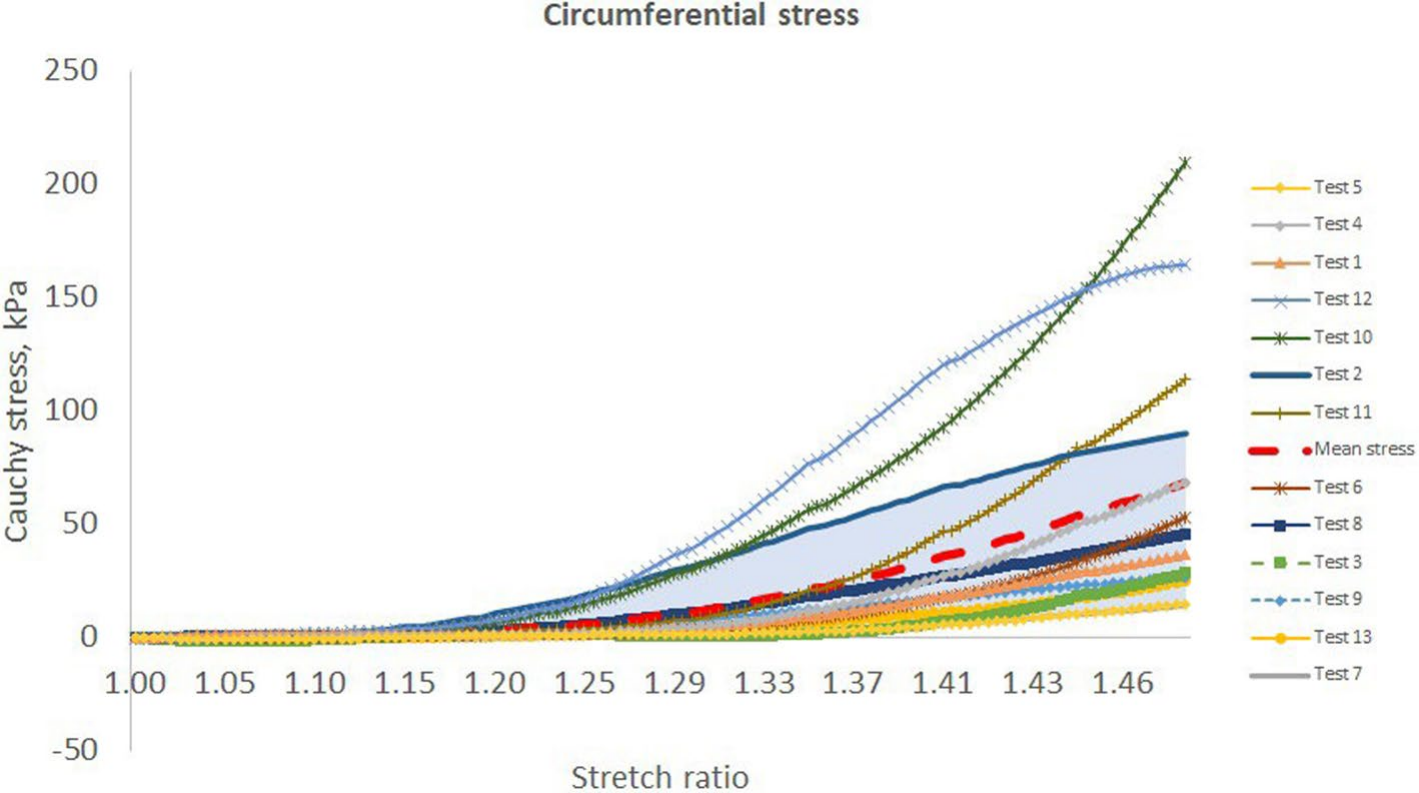
The stretch vs stress curves for the 13 different tests in the study along the circumferential direction. The mean curve is also included. Note the wide diversion of the curves away from the mean graph, especially in the regions of high tissue stretches

In the tested esophageal tissue (Fig. 6 and Fig. 7), it was observed that the toe region extends up to a strain of 40% in both axial and circumferential directions [6]. This is the normal physiological operating range for esophagus. It is reported that esophageal tissue stretches to 50% strain under physiological condition subject to pressures of 3–5 kPa [6]. This strain range affords it the capacity to fulfill its function with ease and comfort.

Figures 6 and 7 show that above 90% of the tests fall within the shaded regions of the stress–stretch ratio graphs. Notice the position of the ‘Mean stress’ curve with respect to the upper and lower bound of the shaded regions. The toe regions almost overlap in both axial and circumferential directions. This implies that the tangential modulus of the test specimens was almost similar. However, the linear regions are spread over a much wider range with modulus of elasticities between 267 and 2671 kPa along the axial direction and between 47 and 1170 kPa along the circumferential direction. All the results lie within a standard deviation of 2.5 to 26.8 kPa for the circumferential stresses as compared with the axial stresses with standard deviations of 4.2 to 27.2 kPa as well as two outlying stress curves at 38.5 kPa and 41.6 kPa. It is not immediately clear whether this could be attributed to lack of tissue preconditioning, insufficient grip at the bio-rake attachment points, animal differences, or even unintended regional differences, which are reported to affect the mechanical properties of the esophageal tissue [6, 42], or whether the differences might have been due to varying testing conditions during those two tests, although the latter may be overruled by the fact that their corresponding circumferential stresses were below 27 kPa.

In the test results, it is further observed that the longitudinal stresses were actually more than two times greater than the circumferential stresses. This is partly in line with the differences between hoop and longitudinal stresses in circular tubes, although it is clear that the esophageal cross-section is not perfectly circular [55]. However, it seems that there is more contribution from the structural differences between the two directions. This difference has to do partly with the different influences of the fiber orientations within the esophageal tissue [4], which should form the subject of further investigation.

### Strain energy

**Fig. 8:**
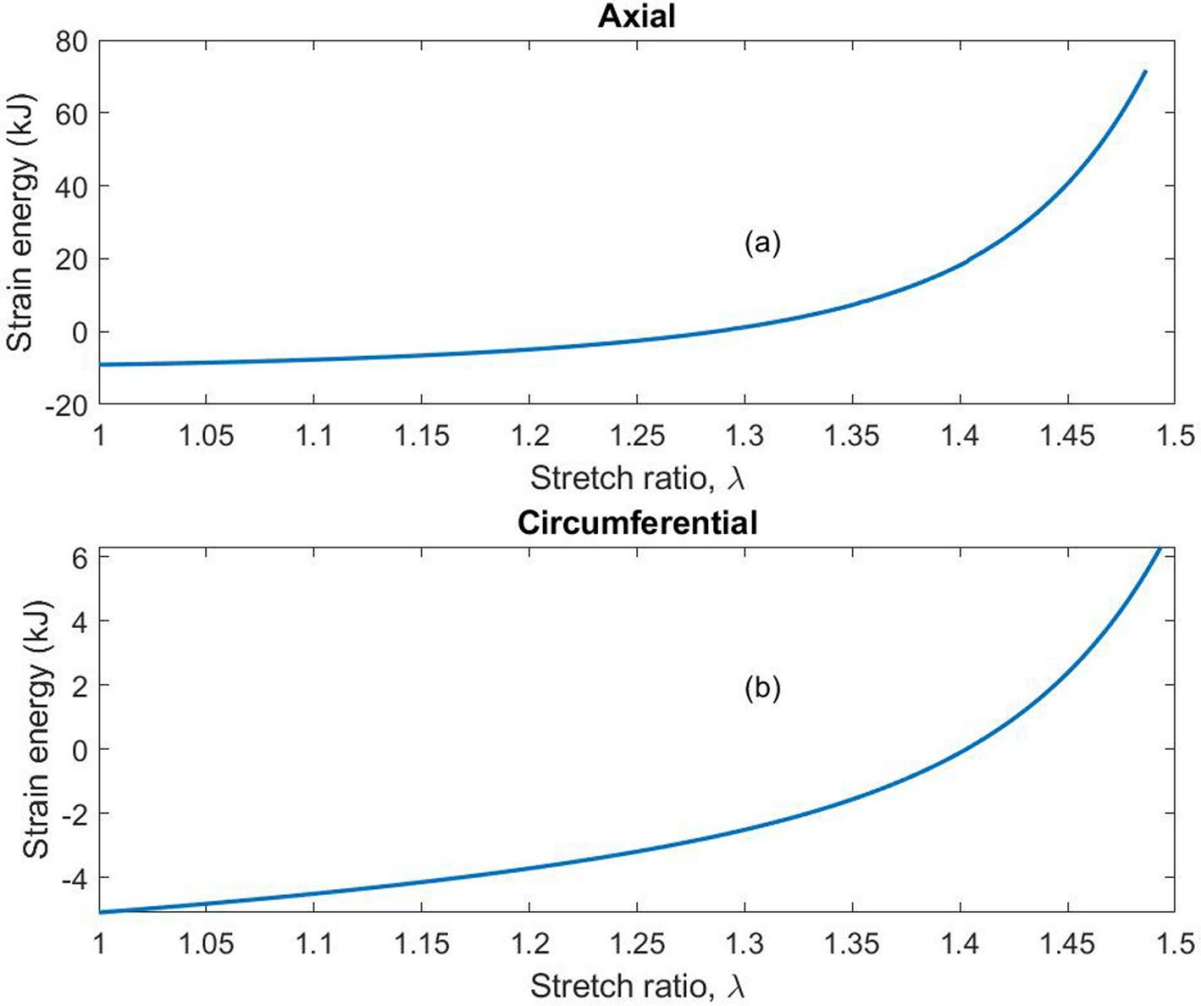
Strain energy functions plotted in the axial and circumferential directions. The circumferential direction was more extensible throughout the tensile testing as shown in **b**, storing much less energy than the axial direction shown in **a**

The strain energy storage in the esophageal tissue was approximated according to the Holzapfel model by Eq. (2). The strain energy along the axial and circumferential directions is shown in Fig. 8. More strain energy is stored in higher stretch ratios than smaller stretch ratios. The average stored energy in the esophagus along the axial direction is approximately 15 kJ, while along the circumferential direction it is approximately 2 kJ. In line with previous studies, the tissue exhibited stiffening at higher stretch ratios, as well as being stronger along the axial direction [6, 7, 43] than along the circumferential direction. It is assumed that collagen fibers do not influence low stress regions such that the toe region tends to be dominated by non-collagenous components [21].

### Results of NARX training performance

**Fig. 9:**
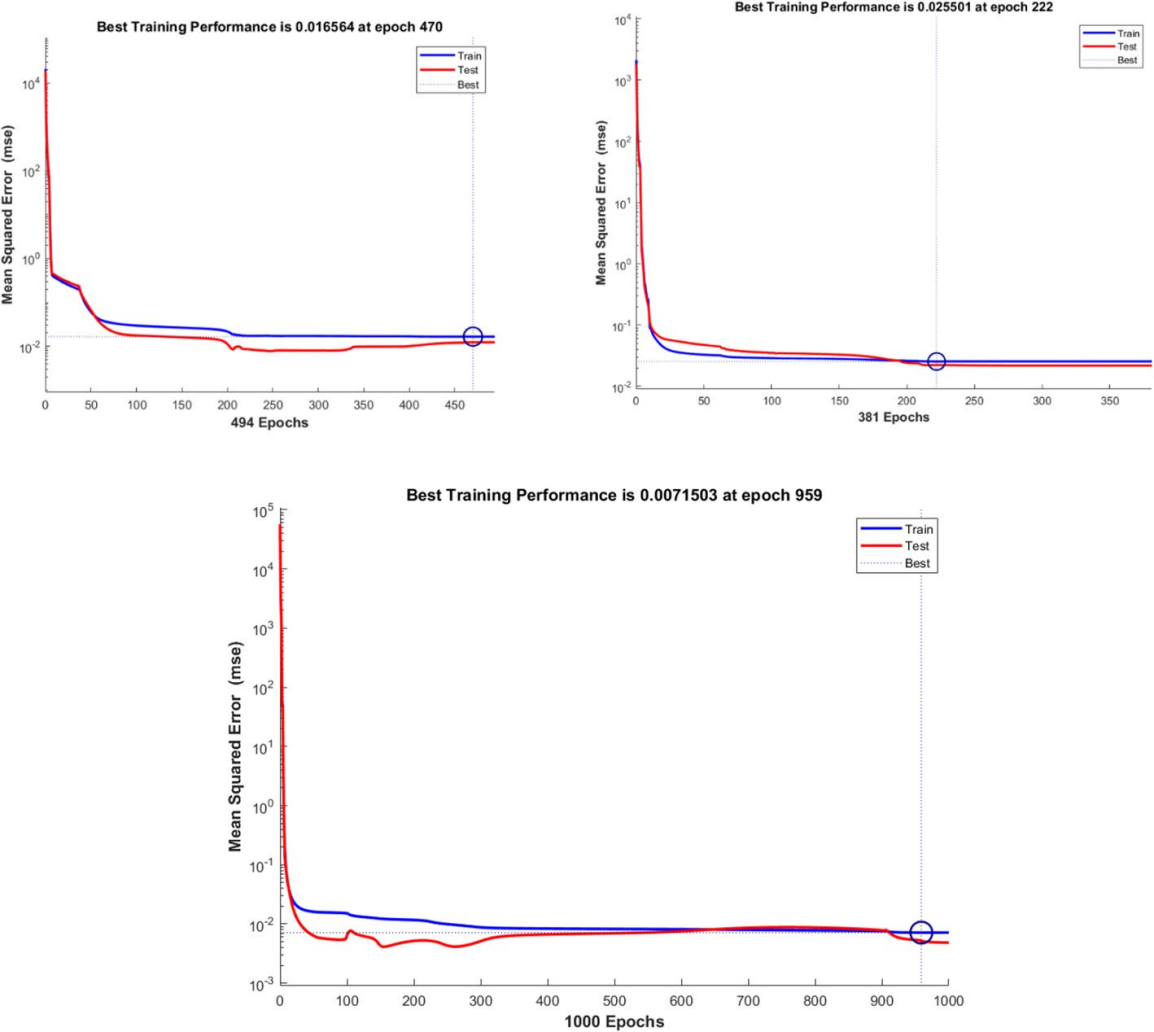
The NARX network performance measured in terms of the minimization of the mean square error value. The ideal convergence point is the mse = 0. The network shows the performance for both the training part (50% of the data) and the test data (25%). It does not show the performance for the validation part (25%)

The following results were obtained after training a 1-5-1 NARX neural network with Test #6 results. The strain data were used as the inputs to the ANN, while stress data were the targets from the ANN. In order to avoid overfitting errors, the neural network was trained with a noise vector which was normally distributed at an amplitude of 1% strain and the Bayesian regularization was employed in the training process. Three different types of training data derived from Test #6 were used: (i) raw test data, (ii) linear curve, and (iii) Holzapfel-type curve. This was done with the aim of demonstrating how feature extraction may aid in the reduction of computational overload and enhance physical interpretation of the artificial neural network results [44, 45]. The three different training curves are shown in Fig. 5 under Sect. "Stress–strain responses". The training parameters for the three curves are summarized in Table 1. Notice that the training stopping condition for the three forms of training data were similar for the raw test and linear curve-fitted data. Both processes stopped after reaching maximum $\mu$, which implied that no further improvements were possible unless more processing neurons were added to the hidden layer. However, the Holzapfel model-fitted data show that the network converged after attaining $\mu =0.5$, which gives room for further improvement by simply changing the training data. The training processes were stopped at 494 for the raw test data and at 381 for the linear curve-fitted data out of a possible default setting of 1000 training epochs. Both processes completed the training procedure in less than 1 s. The performance criterion which was measured by mse was reduced to 0.0166 for the raw test data, 0.0255 for the linear curve-fitted data, and 0.00716 for the Holzapfel model-fitted data from a possible default setting of $mse=0.$ The gradient of the objective function (mse) was reduced to 0.00391 for the raw test data, 0.00583 for the linear curve-fitted data, and 0.00195 for the Holzapfel model-fitted data from a possible gradient equal to zero. It is shown that although the Holzapfel model-fitted data yielded more accurate results than the other two training data, the neural network converged at 1000 training epochs, of course this was rounded up from the exact number of epochs of 959, as observed from the performance curves in Fig. 9.

**Table 1:** The training data and how the NARX network performed using each of the data sets

Network training parameters	Training data and parameter values		
	Test data	Linear curve fitted	Holzapfel model-fitted curve
Epoch	494	381	**1000**
Time	< 1 s	< 1 s	1 s
Performance	0.0166	0.0255	0.00716
Gradient	0.00391	0.00583	0.00195
Mu	**5 × 10**^**5**^	**5 × 10**^**5**^	0.05
Effective number of parameters	22.3	19.5	22.9
Sum of squared parameters	6.09	4.63	5.76

The training process for test data and linear curve-fitted data was stopped due to the network reaching the maximum Mu. The Holzapfel model-fitted data stopped the network training after reaching the maximum number of epochs

The advantages of using the Bayesian regularization lie in what is shown for the “Effective number of parameters” and “Sum of squared parameters”. Notice that from the default settings of 31 effective number of training parameters and approximately 36 sum of squared parameters, the regularized network converged to 22.3, 19.5 and 22.9 effective parameters and 6.09, 4.63 and 5.76 Sum of squared parameters for the raw test data, linear curve-fitted data and Holzapfel model-fitted data, respectively. The result of these reductions in the effective parameters of the network is a corresponding gain in computational efficiency without a loss in network accuracy. Thus, despite the best fitting accuracy of the Holzapfel model-fitted data, the linear curve fit such as the neo-Hookean model-fitted data may still afford the neural network less computational effort in the approximation of esophageal tissue tensile behavior. This is also evident in the relatively faster training durations of the linear curve-fitted data as compared with the Holzapfel model-fitted data in Table 1. The training data and how the NARX network performed using each of the data sets. The training process for test data and linear curve-fitted data was stopped due to the network reaching the maximum Mu. The Holzapfel model-fitted data stopped the network training after reaching the maximum number of epochs.

**Fig. 10:**
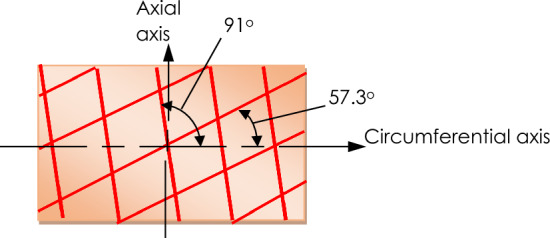
Diagrammatic representation of the fiber orientation as calculated by the Holzapfel model. The angles are measured from the axis along the circumferential direction

**Fig. 11:**
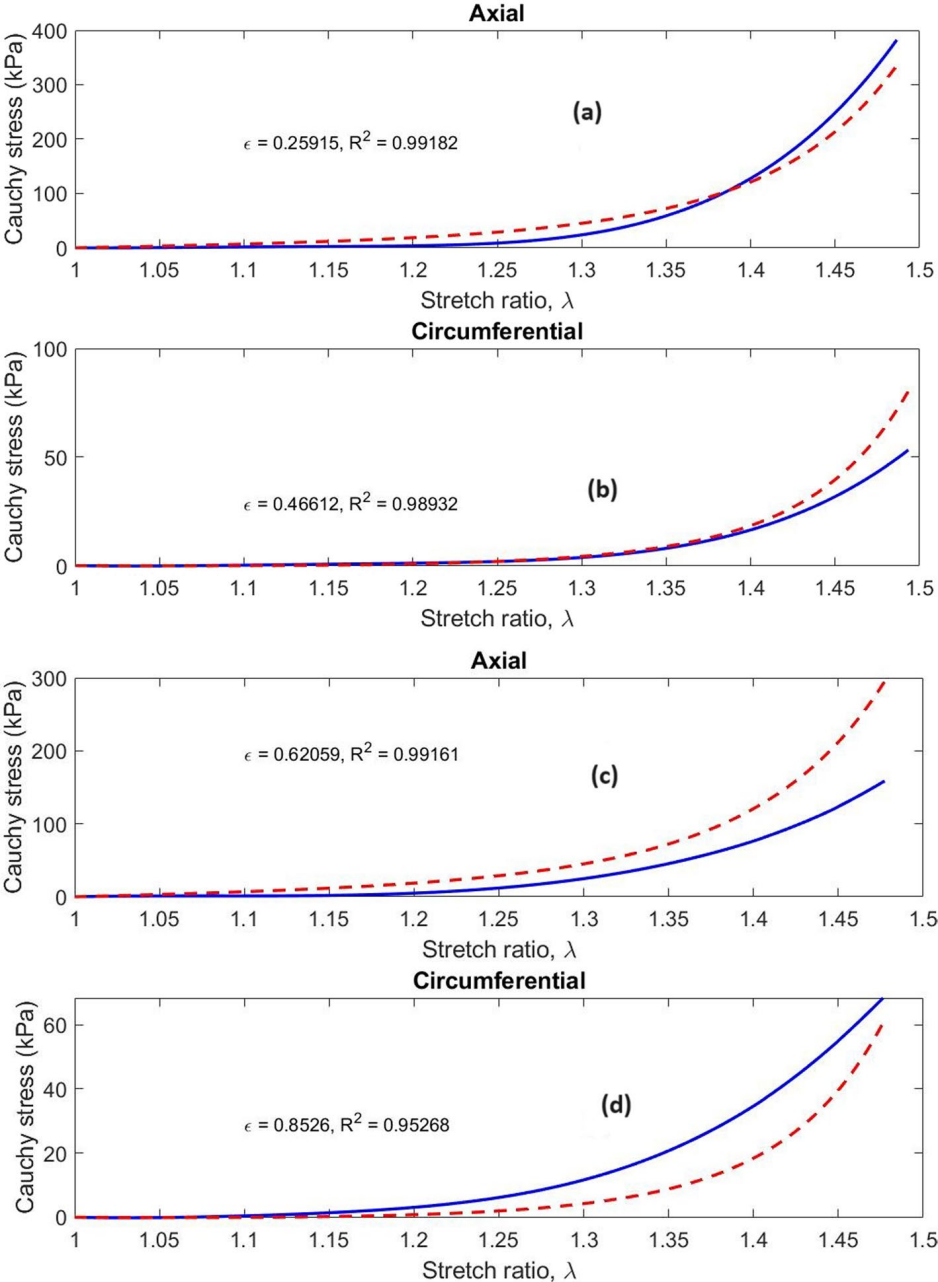
Typical Cauchy stress vs stretch curves for the esophageal tissue along the axial and circumferential directions estimated through the application of the Holzapfel model. The solid lines show the experimental results, while the dashed lines show the Holzapfel model estimated curves. Graphs (**a**) and (**b**) correspond to Test 6 while Graphs (**c**) and (**d**) are for Test 13. Each graph shows the fitting error, ε value calculated through the normalized root mean square error and the R^2^ value calculated via the coefficient of determination

**Fig. 12:**
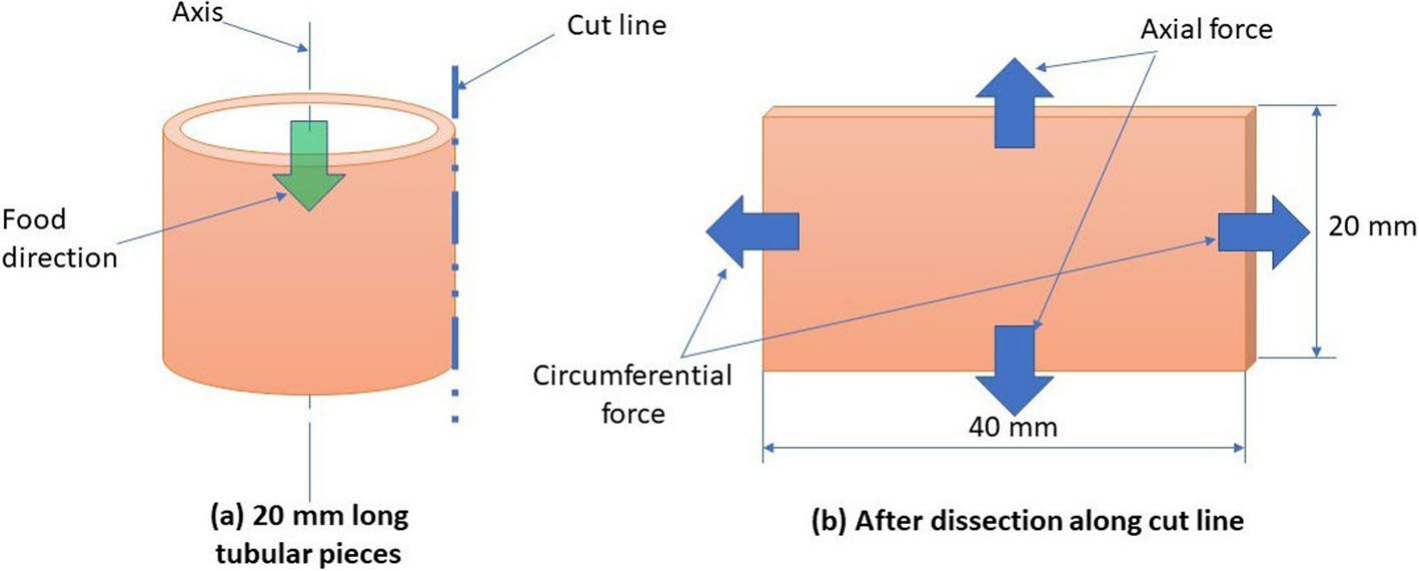
Schematic diagrams of the test pieces. **a** Represents the 20-mm-long tubular specimens cut along the food direction. This specimen was further sliced through the cut line to obtain final specimen (**b**). The resulting 20 mm by 40 mm specimen was mounted on a biaxial testing machine

**Fig. 13:**
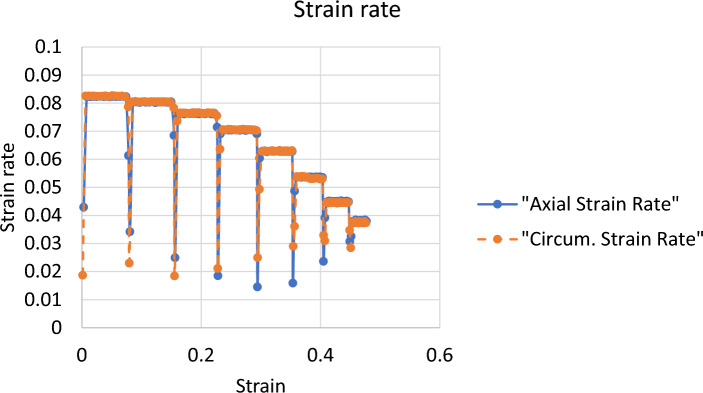
Strain rate evolution during the biaxial tensile testing of the esophageal tissue. The strain rates did not remain constant at one level; as the test progressed the level decreased from slightly above 8%/s to slightly below 4%/s. The strain rates were calculated from the average curves

**Fig. 14:**
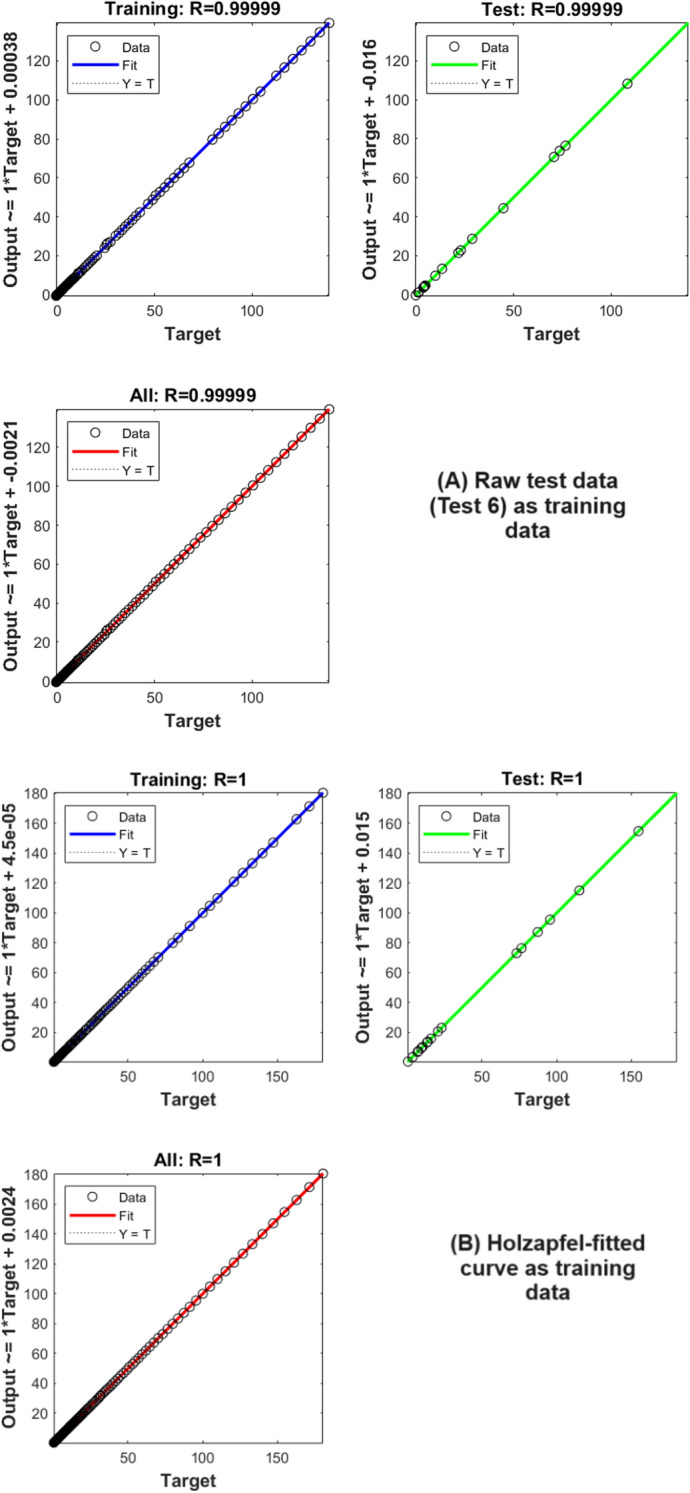

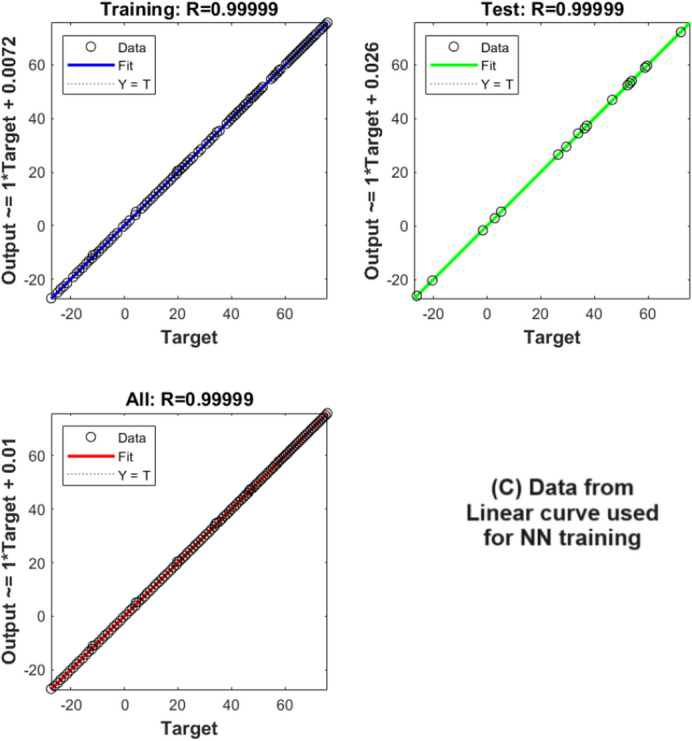
The regression performance of the NARX network for the three data divisions/parts: training (50%), validation (25%), and test (25%). **A** Presents the performances for these three data parts for the raw test data only. **B** Presents the performance for these three parts for the Holzapfel model-fitted data, and **C** presents the performance for the linear curve-fitted data

The results of the regression performance for the training part (50%), validation part (25%), and test part (25%) of each type of training data are presented in Figure 14 in the Appendices. All the parts show very high correlation R at percentages greater than 99.9%. The fact that the Holzapfel model-fitted data return correlations R equal to 100% on all three data divisions/parts is noteworthy.

When the trained NARX was tested/simulated on fresh sets of data, the results in Table 2 show that the linear curve-fitted data yielded the largest fitting error of approximately 32% on the circumferential stress on Test #5, whereas the smallest error was obtained on Test #12 at the fitting error of 4.4% for the circumferential stress. The values in bold in the table show the lowest and highest fitting errors along each direction for the three different forms of training data. Overall, the Holzapfel model-fitted data produced the most accurate results, with fitting errors averaged at approximately 11% and 10% along the axial and circumferential directions, respectively. The linear curve-fitted data yielded the largest average fitting errors at approximately 13% and 16% along the axial and circumferential directions, while the raw test data had approximately 13% and 11% along the axial and circumferential directions. In terms of correlations, all the training data forms were accurate to a minimum correlation coefficient of 99.9%.

**Table 2:** A comparison of the fitting error and correlation coefficients for the three different types of training data

	Holzapfel—NARX				Linear—NARX				Raw measured data—NARX			
Test Data	Fit errors		Corr. coeff.		Fit errors		Corr. coeff.		Fit errors		Corr. coeff.	
	Axial	Circ.	Axial	Circ.	Axial	Circ.	Axial	Circ.	Axial	Circ.	Axial	Circ.
T1	0.0789	0.0889	0.9999	0.9999	0.1181	0.1427	0.9997	0.9997	0.1294	0.1084	0.9998	0.9998
T2	0.0977	**0.0698**	1	0.9999	0.069	0.0536	0.9998	0.9998	0.099	**0.076**	0.9997	0.9998
T3	**0.143**	**0.1566**	0.9995	0.9995	**0.2819**	0.2801	0.9987	0.9991	**0.1921**	**0.175**	0.9995	0.9991
T4	0.1223	0.1148	0.9998	0.9999	0.176	0.1259	0.9995	0.9996	0.1711	0.1275	0.9997	0.9997
T5	0.1404	0.1283	0.9999	0.9997	0.21	**0.3181**	0.9995	0.9993	0.1725	0.1384	0.9997	0.9997
T6	0.1387	0.1203	1	1	0.0699	0.1531	0.9994	0.9999	0.1236	0.1418	0.9999	0.9999
T7	0.1245	0.1004	0.9998	0.9998	0.2078	0.3059	0.9996	0.9992	0.1588	0.1135	0.9998	0.9998
T8	0.1384	0.0738	1	0.9999	0.0806	0.1088	0.9999	0.9998	0.1271	0.0868	0.9999	0.9999
T9	0.1098	0.071	0.9999	0.9999	0.1851	0.1551	0.9996	0.9998	0.146	0.0975	0.9998	0.9998
T10	0.0871	0.1091	0.9999	1	0.057	0.0524	0.9999	0.9991	0.0883	0.1037	0.9998	1
T11	0.0933	0.1165	0.9999	0.9999	0.0787	0.0919	0.9997	0.9998	0.1064	0.1229	0.9997	0.9998
T12	0.0784	0.0914	0.9997	0.9999	**0.0505**	**0.0435**	0.9994	0.9997	**0.0784**	0.084	0.9992	0.9998
T13	**0.0753**	0.0977	0.9998	0.9999	0.0546	0.2198	0.9997	0.9997	0.0837	0.113	0.9996	0.9999
Mean	**0.1098**	**0.1030**			**0.1261**	**0.1578**			**0.1290**	**0.1145**		

On average, the Holzapfel-fitted curve shows the highest fitting accuracy, with most of the correlation coefficients being close to 100%

In Table 3, the analysis of variance (ANOVA) was conducted to assess the differences in the results obtained by using the three different forms of training data. The results show no statistical significance in the use of one form of training data form over another. This shows that one can use any one of the three forms of training data if the main interest is merely to assess the tensile behavior of the esophageal tissue. However, if the researcher needs to extract some important material parameters, then the use of the linear curve and Holzapfel model-fitted curves is necessary. It should be pointed out that the use of the pure linear curve has to be substituted by the neo-Hookean model representation if physically meaningful material parameters that are useful for finite element modeling are desired [35, 46].

**Table 3:** Evaluation of the variances among the three different types of training data. Single-factor ANOVA was conducted for both axial and circumferential stresses

ANOVA: single factor for axial stresses							ANOVA: single factor for circumferential stresses						
Summary							Summary						
*Groups*	*Count*	*Sum*	*Average*	*Variance*			*Groups*	*Count*	*Sum*	*Average*	*Variance*		
Column 1	13	1.4278	0.1098	0.0007			Column 1	13	1.3386	0.1030	0.0006		
Column 2	13	1.6392	0.1261	0.0059			Column 2	13	2.0509	0.1578	0.0091		
Column 3	13	1.6783	0.1291	0.0013			Column 3	13	1.8018	0.1386	0.0013		
ANOVA							ANOVA						
*Source of variation*	*SS*	*df*	*MS*	*F*	*P-value*	*F crit*	*Source of variation*	*SS*	*df*	*MS*	*F*	*P-value*	*F crit*
Between groups	0.0028	2	0.0014	0.5367	**0.5893**	3.2594	Between groups	0.0201	2	0.0101	2.7263	**0.0790**	3.2594
Within groups	0.0937	36	0.0026				Within groups	0.1327	36	0.0037			
Total	0.0965	38					Total	0.1528	38				

The analyses of variances were conducted on the fitting errors only. It was assumed that the correlation coefficients would yield similar results

A further observation relating to the performance of the neural network is that it returned more accurate predictions in the toe region than in the linear region. Most analytical and computational models do not yield such accuracies within the toe region due to gradient issues within this region. Apparently, this could be attributed to the proximity of the stress–strain curves to one another within this region, as opposed to the widely dispersed linear region.

Another observation is that the ANN yields more accurate fitting and correlation percentages for circumferential stresses than for longitudinal stresses except for the linear curve-fitted training data. The spread of the curves in the circumferential stresses is narrower than in the longitudinal stresses. Thus, it is relatively easier for the neural network to produce more accurate results due to the narrower margins within the search region [44, 45]. On the other hand, many of the difficulties relating to the linear curve-fitted training data can be attributed to the difficulties in predicting the circumferential stresses for Tests #3, #5, and #7, which belonged to the ‘Poor training data’ class. It is thus clear that in such cases, the linear curve-fitted data did not contain sufficient features to estimate these test data accurately.

Although Test #6 was the only one that was used to derive and construct all forms of training data used in this study, the trained neural network was able to produce high fitting accuracies and correlation coefficients without overfitting any of the stress curves. This demonstrated a great deal of network generalization. The neural network attained a good enough level of generalization with a noise amplitude of 1 kPa, and it was qualitatively observed that more noise at 10 kPa did not produce any improvements in the network results.

### Use of different training data

Another equally important factor was the selection of the training set. Through the trial-and-error method, it was shown that other training sets such as Test #3 yielded very poor results compared with Test #10. In Sect. "Stress–strain responses", data were classified into two groups depending on their training performance. ANOVA testing of the training data from the two different classes showed that the differences were statistically significant [38]. In Table 4(A) the evaluation of differences in the correlation coefficients between Tests #3 and #10 yielded a P-value of 0.000126, while in Table 4(B) the same analysis between Tests #7 and #14 produced a P-value equal to 0.00092. In the test of significance the differences are significant if the P-value is less than 0.05[40]; thus the results obtained here show that the two groups of data sets are not equal. Thus, the choice of training data is a very important factor in neural network training. This is especially important where raw test data are used directly as training data without due consideration for feature identification and extraction. Such considerations are crucial where neural networks are used for function approximation [44, 45].

**Table 4:** The ANOVA for four types of training data drawn from the two different classes of training data

A										
Test 3		Test 10		ANOVA: single factor						
Correlation coeff.										
Axial	Circ.	Axial	Circ.	Summary						
0.9449	0.9987	0.9997	0.9997	*Groups*	*Count*	*Sum*	*Average*	*Variance*		
0.8496	0.9328	0.9997	0.9998	Column 1	13	11.0045	0.8465	0.014059		
0.9689	0.9835	0.9988	0.9992	Column 2	13	11.9899	0.9223	0.018762		
0.9927	0.9993	0.9993	0.9996	Column 3	13	12.985	0.998846	2.82E-06		
0.7466	0.9843	0.9995	0.9997	Column 4	13	12.9961	0.9997	3.33E-08		
0.9833	0.9996	0.9966	0.9999							
0.6658	0.9995	0.9996	0.9997							
0.9908	0.9998	0.994	0.9998	ANOVA						
0.7247	0.5424	0.9996	0.9998	*Source of Variation*	*SS*	*df*	*MS*	*F*	*P-value*	*F crit*
0.8153	0.8014	0.9995	0.9996	Between groups	0.209	3	0.069632	8.485699	**0.000126**	2.798061
0.7739	0.779	0.9994	0.9996	Within groups	0.394	48	0.008206			
0.7073	0.9997	0.9996	0.9999							
0.8407	0.9699	0.9997	0.9998	Total	0.603	51				

B										
Test 7		Test 14		ANOVA: SINGLE factor						
Correlation coeff.										
Axial	Circ.	Axial	Circ.	Summary						
0.9987	0.9619	0.9998	0.9996	*Groups*	*Count*	*Sum*	*Average*	*Variance*		
0.9747	0.8413	0.9997	0.9995	Column 1	13	12.1684	0.936031	0.007723		
0.9993	0.9929	0.9993	0.9992	Column 2	13	11.3009	0.8693	0.021666		
0.998	0.8966	0.9994	0.9996	Column 3	13	12.9649	0.9973	3.63E-05		
0.9997	0.9994	0.9996	0.9996	Column 4	13	12.9413	0.995485	0.000122		
0.8019	0.9532	0.9914	0.9999							
0.761	0.9359	0.9996	0.9998							
0.9997	0.9952	0.9787	0.9998	ANOVA						
0.9219	0.5283	0.9997	0.9998	*Source of variation*	*SS*	*df*	*MS*	*F*	*P-value*	*F crit*
0.9791	0.7472	0.9993	0.9619	Between groups	0.143	3	0.047722	6.46046	**0.00092**	2.798061
0.9578	0.6868	0.9997	0.9993	Within groups	0.355	48	0.007387			
0.8	0.9942	0.999	0.9834							
0.9766	0.768	0.9997	0.9999	Total	0.498	51				

(A) conducts ANOVA between Test 3 from Poor Training Data and Test 10 from Good Training Data. (B) conducts ANOVA between Test 7 from Poor Training Data and Test 14 from Good Training Data

### Stress–strain responses

The following two sections report the stress responses after the Holzapfel model and the NARX network were applied to the new test data.

#### Holzapfel model results

The estimations of the Cauchy stresses based on the Holzapfel model as given in Eqs. (4) and (5) are presented in this section simply as a way of appreciating the application and performance of the NARX model in the next section. In calculating the Cauchy stresses from the Holzapfel model, the model was initially fitted to Test #8 stress–strain curves to determine the material parameters $c, {k}_{1}, {k}_{2},$ and $\theta$. Test #8, just like Test #6 belonged to the ‘Good training data’. A nonlinear least squares estimator employing the Levenberg–Marquardt (LM) algorithm was used in the optimization routine. The advantage of using the LM algorithm is that it approximates second order gradients through first order approximation and ensures that the optimization process is well-conditioned throughout [44, 45]. For the biaxial tensile test in the study, the global minimum for the sum of squared errors in both axial and circumferential directions was sought as given in Eq. (6). Different material parameters were obtained along the two test directions. The relevant algorithms were implemented in MATLAB. The choice of the Holzapfel model was based on its capability to model multi-layered tissue similar to the esophagus [18, 22, 47, 48]. The material parameters are presented in Table 5.

**Table 5:** Material parameters for the esophagus tissue calculated using the Holzapfel model along the axial and circumferential direction

Material parameter	Axial direction	Circumferential direction
$c$	9.0966	5.0863
${k}_{1}$	9.3158	1.1122
${k}_{2}$	1.8472	1.7420
$\theta$	1.0002	1.5884

The parameters *c* and *k*_1_ are stress-like parameters, *k*_2_ is a dimensionless parameter, and $\theta$ is the angle between the collagen fiber orientation and the circumferential direction

The fiber orientation $\theta$ in the axial direction is equivalent to 57.3° and 91° in the circumferential direction (see schematic in Fig. 10) merely for demonstration purposes.

Typical results of fitting the Holzapfel model to the rest of the experimental data are presented in Fig. 11. Model estimations along the circumferential and axial directions are plotted over experimental biaxial test results in the figure. The plotted results show that model estimated results correlate well with experimental results at above 95% correlation. Some of the tests have correlations above 99%. The fitting errors are, however, quite high due to the inaccuracies of the model at higher ranges of stretch ratio. This could be caused by model deficiencies in incorporating shear effects and in modeling the effects of collagenous and adipose tissue of the esophagus whose effects tend to dominate the higher stress regions [22]. In the tissue preparation process, no effort was made to remove the adventitia, which might not be sufficiently captured by the anisotropic part of the Holzapfel model. The plotted results do not really show that the model was particularly more accurate in stress prediction along a specific direction.

#### NARX stress–strain responses

In this section, the results of the NARX simulations on the 13 biaxial tensile test results are reported. The NARX neural network was implemented in MATLAB. In order to have proper control of both the training and simulation processes the code was custom-written, thus avoiding the blind use of a neural network toolbox in MATLAB. For the results presented in this section, the training data were derived from fitting the Holzapfel model to Test #6 data as shown in Fig. 5 under Sect. "Stress–strain responses". As observed from the fitted curve, it is not a perfect fit to the test data, although it provides the advantage of being able to interpret it in terms of physical material parameters such as the esophageal tissue tangential modulus, linear stiffness, and fiber orientation in the axial and circumferential directions.

**Fig. 15:**
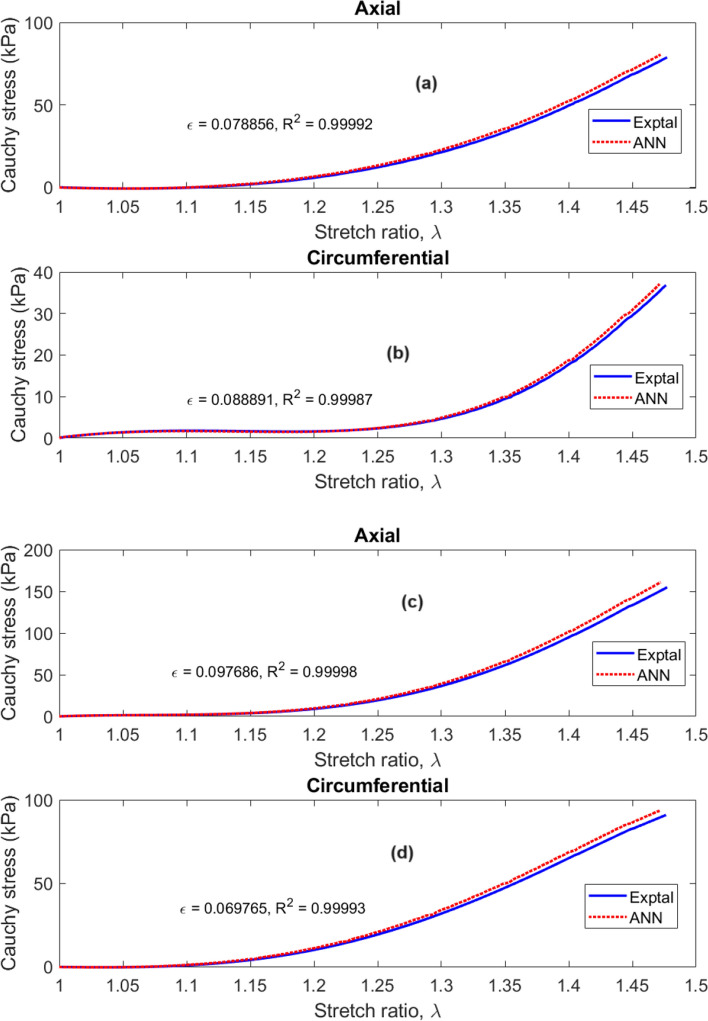

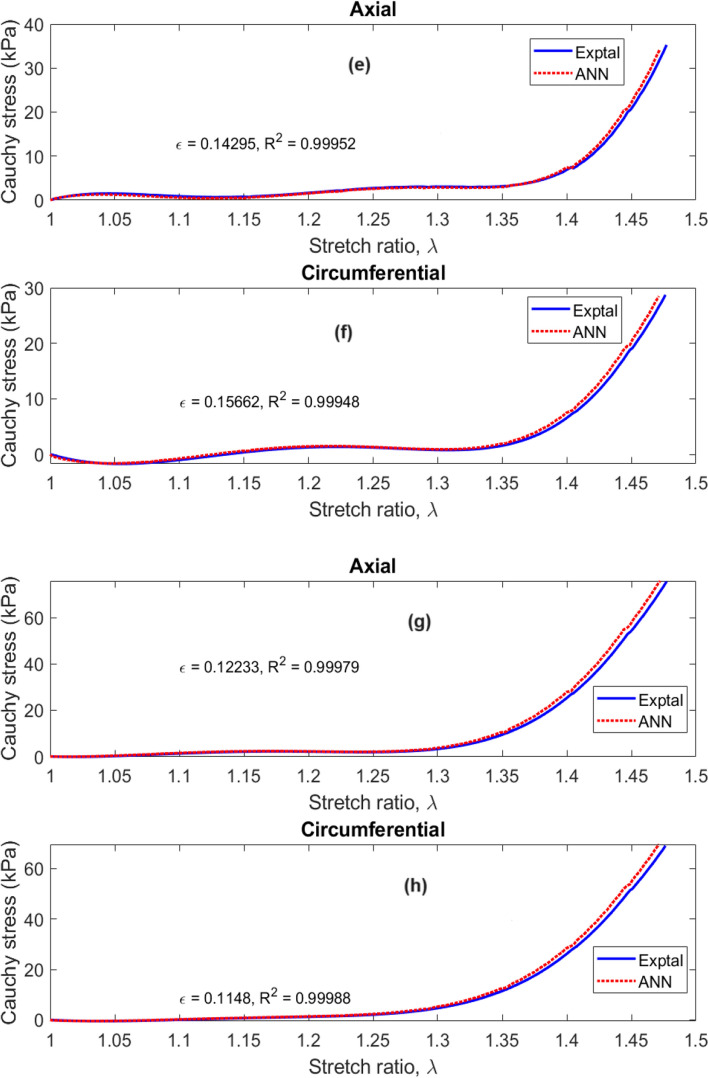

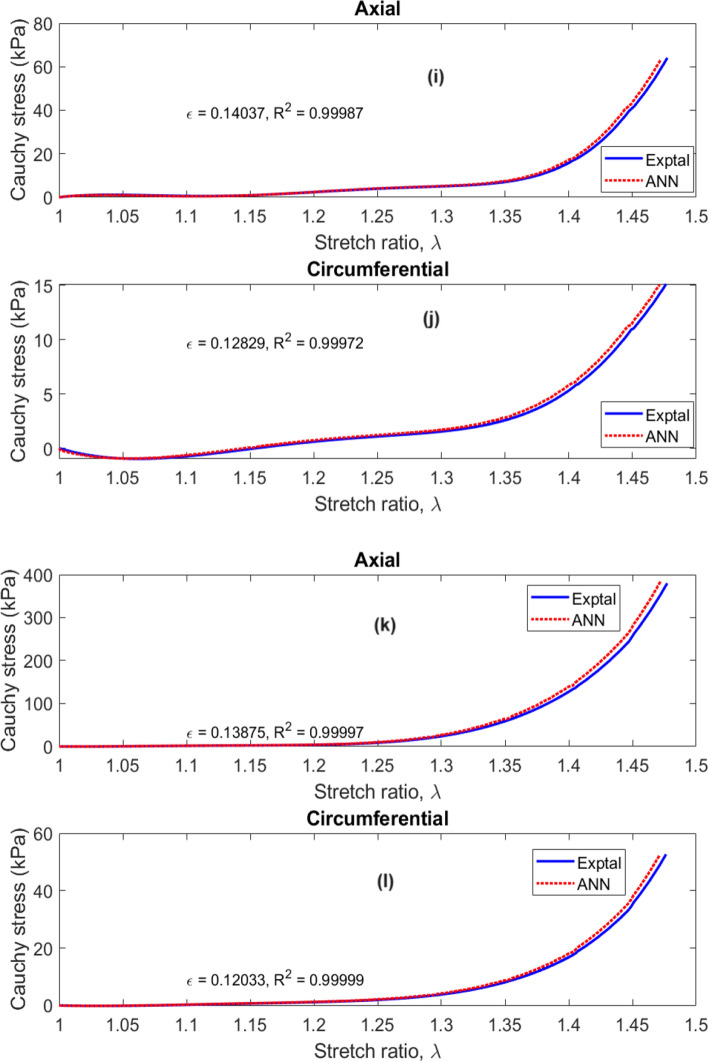

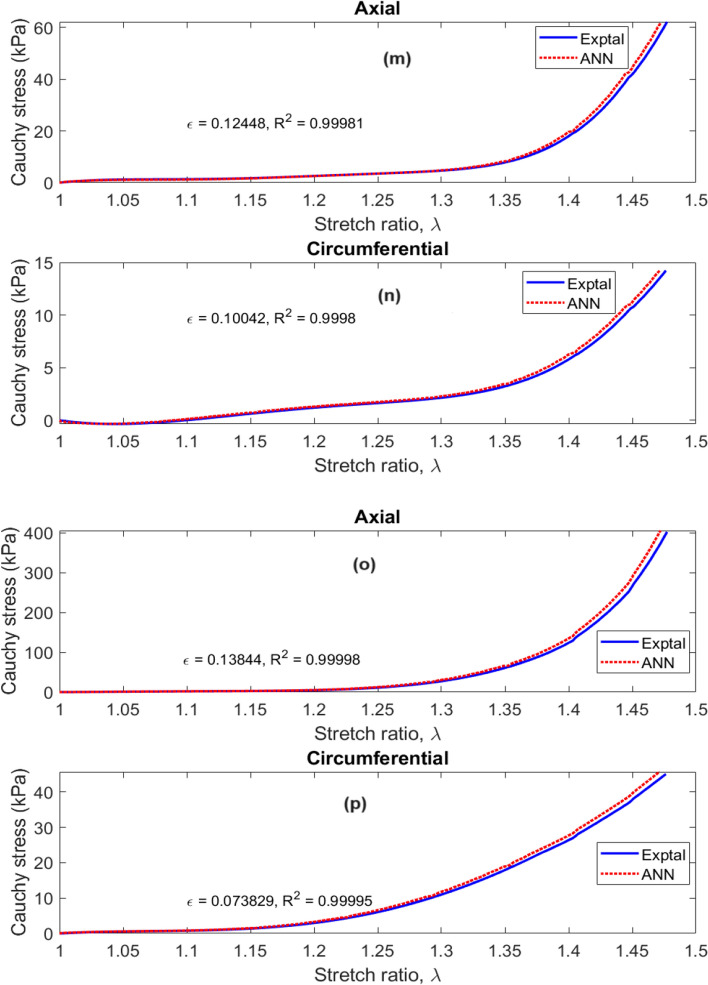

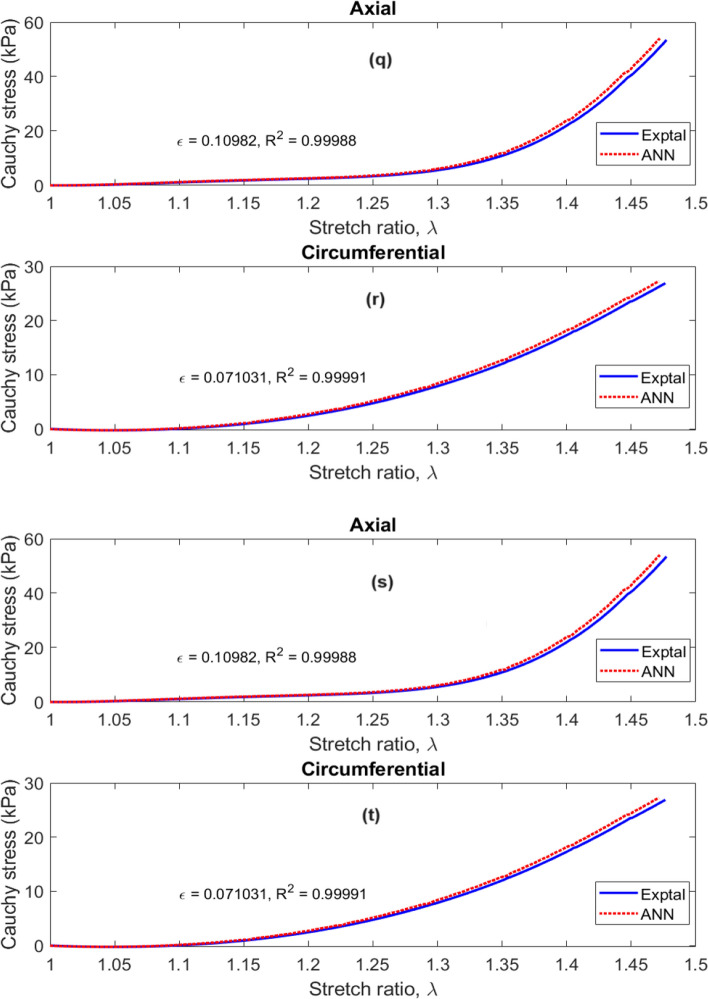

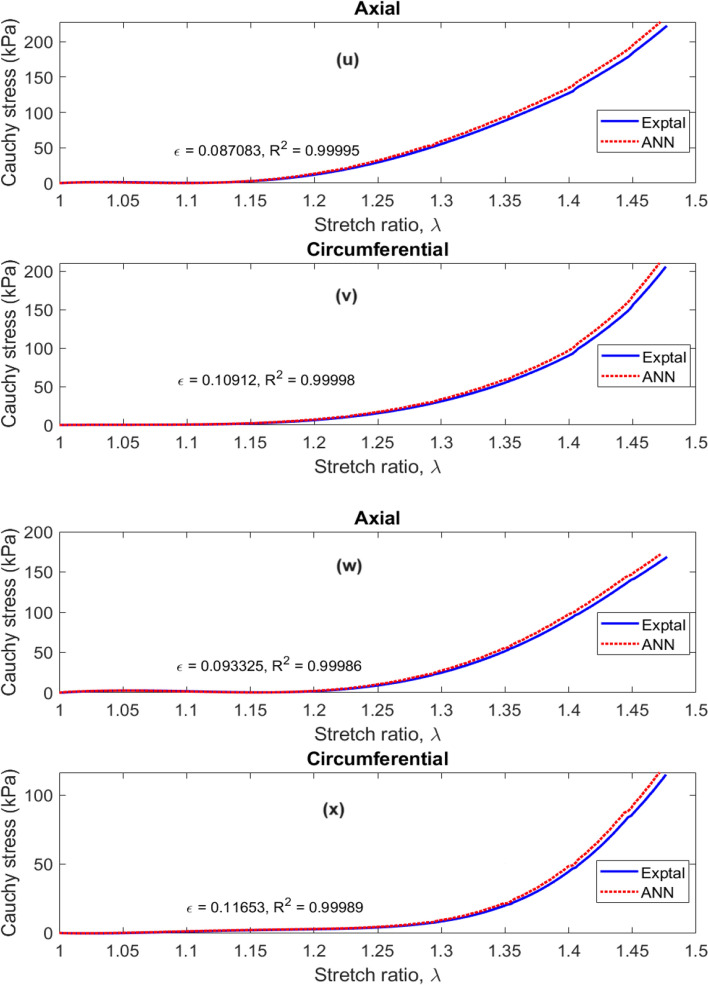

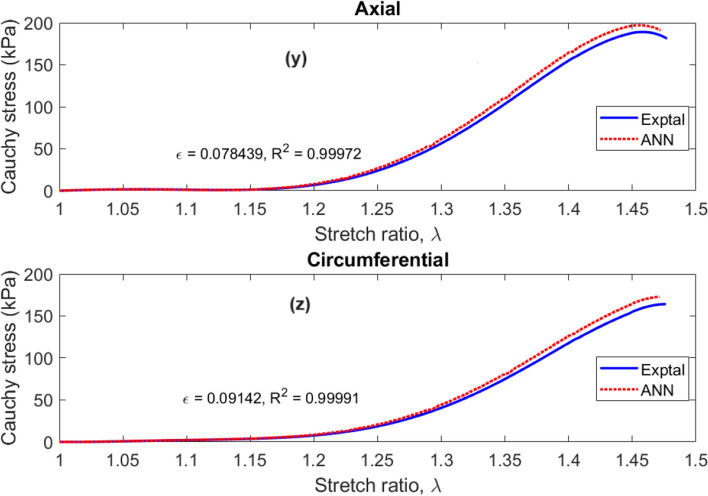
NARX network stress–stretch ratio curves plotted over experimentally measured curves for 13 test results. The plotted graphs are for the following tests: **a** and **b—**Test 1; **c** and **d—**Test 2; **e** and **f**—Test 3; **g** and **h**—Test 4; **i** and **j**—Test 5; **k** and **l**—Test 6; **m** and **n**—Test 7; **o** and **p**—Test 8; **q** and **r**—Test 9; **s** and **t**—Test 10; **u** and **v**—Test 11; **w** and **x**—Test 12; and **y** and **z**—Test 13

The NARX network was simulated on the test data and the corresponding results were plotted over experimentally measured results in Figure 15 in the Appendices. For each test, stresses in the axial and circumferential directions are presented. The NARX network predicted Cauchy stresses are accurate to within 16% fitting error at the correlation level of greater than 99.9%. As shown, the NARX results closely match the different stress curves despite their being widely dispersed at large tissue stretches. The NARX results are clearly more accurate than the conventional Holzapfel model results reported in Sect. Holzapfel model results.

**Fig. 16:**
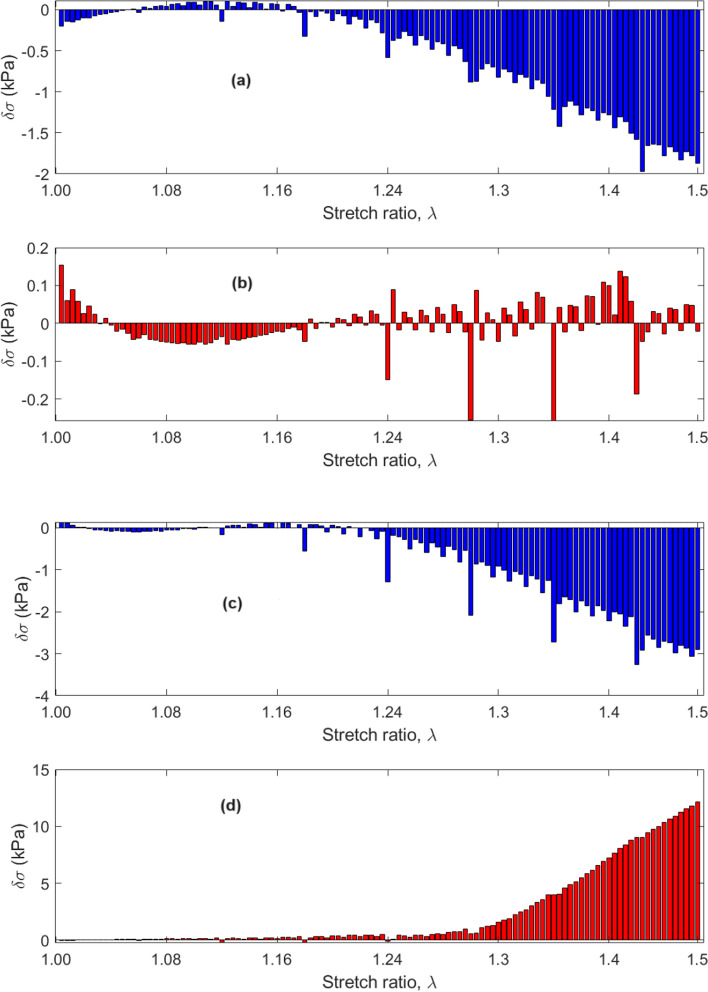

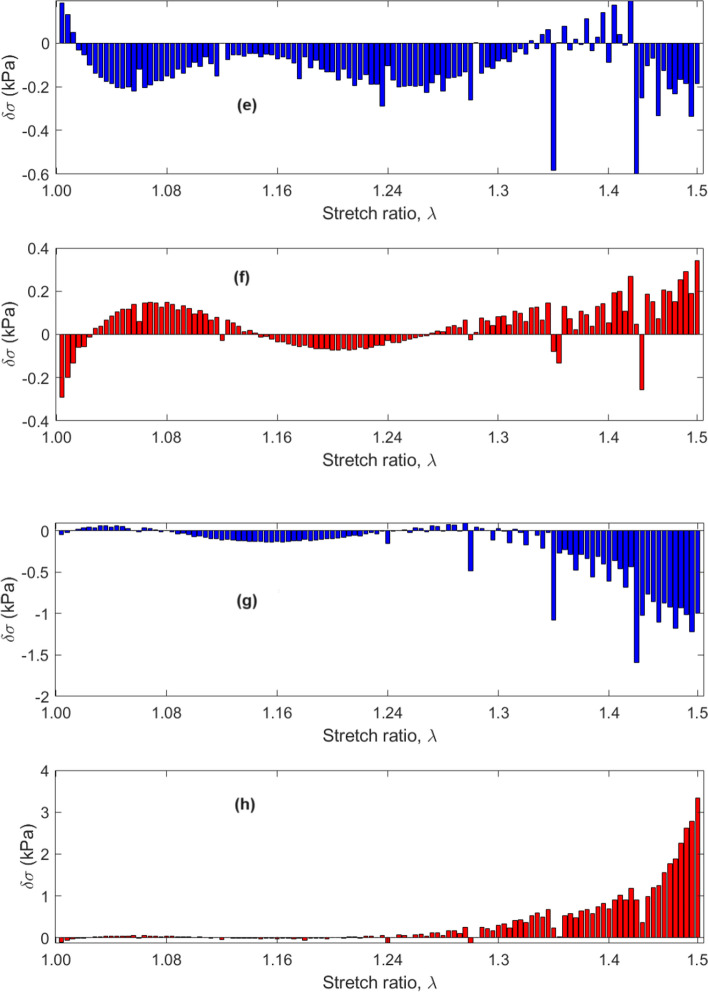

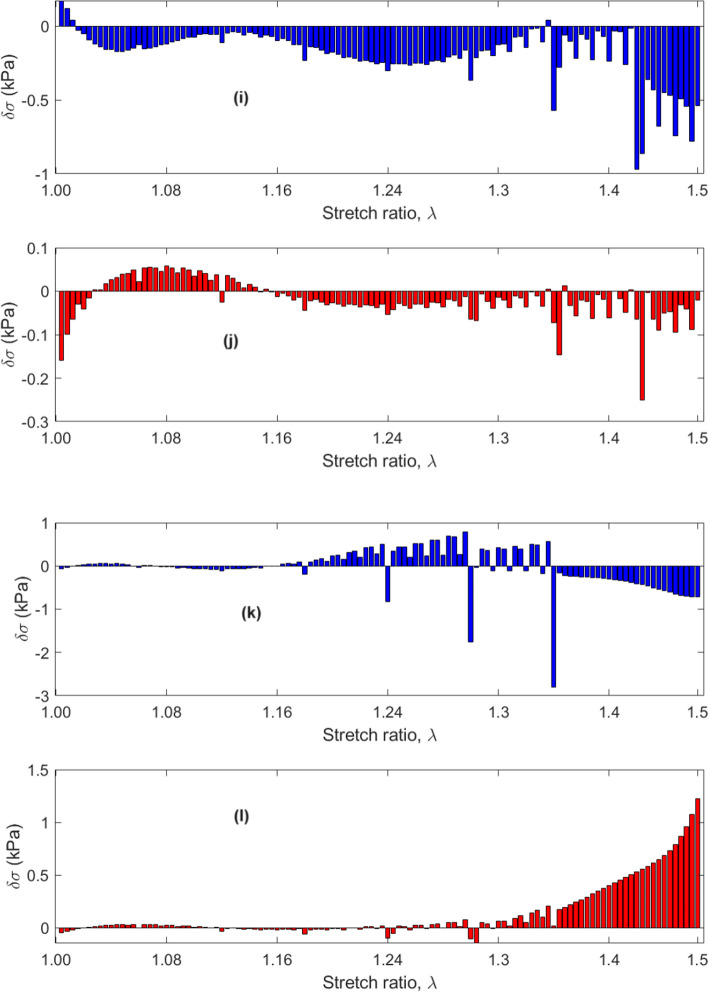

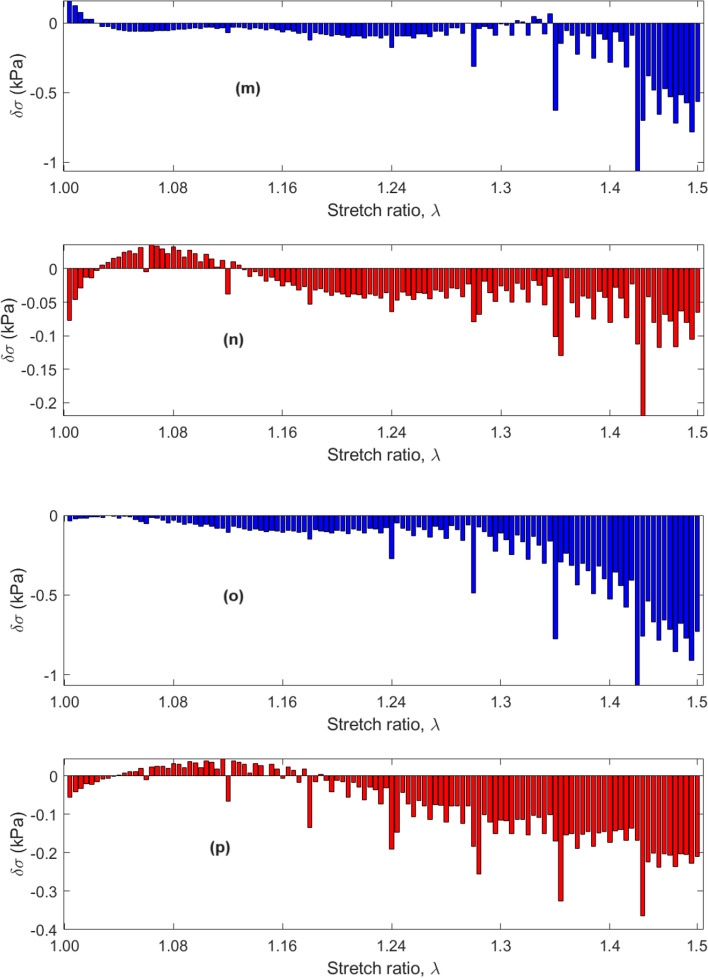

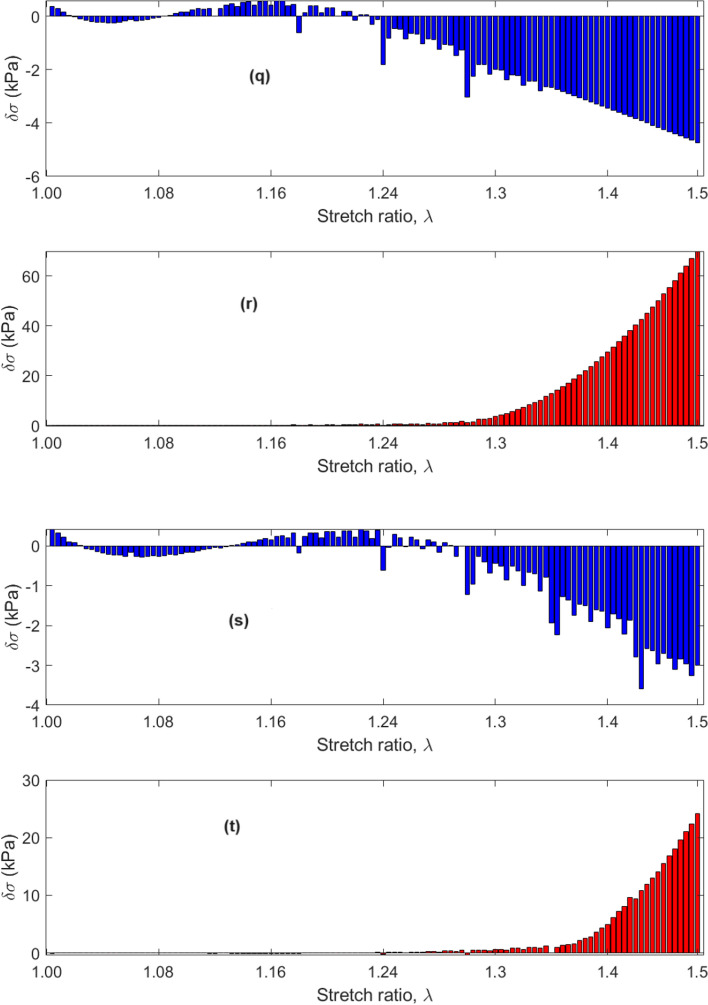

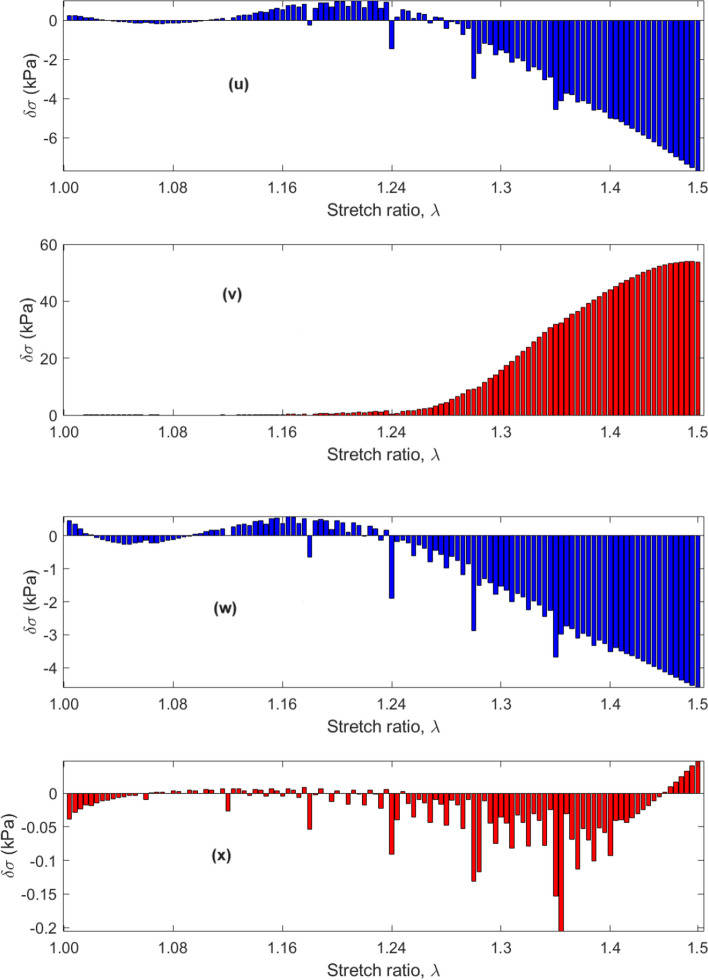

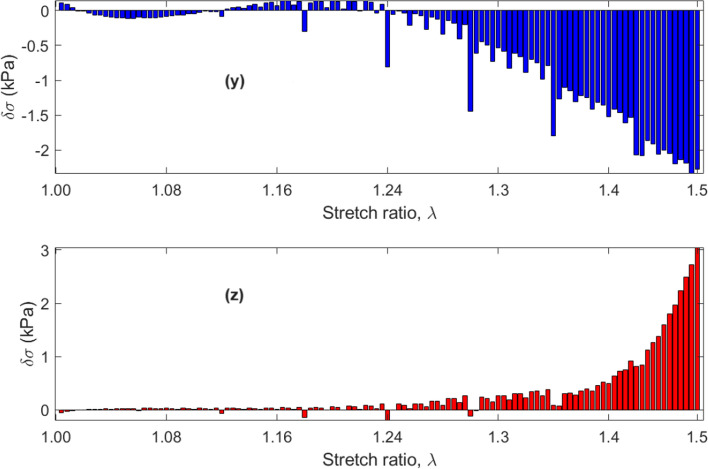
Stress error plots of the ANN predictions in the axial (**a**, **c**, **e**, **g**, **i**. **k**, **m**, **o**, **q**, **s**, **u**, **w**, and **y**) and circumferential (**b**, **d**, **f**, **h**, **j**, **l**, **n**, **p**, **r**, **t**, **v**, **x**, and **z**) directions. Most plotted errors show larger values at the higher strain values. The plotted graphs are for the following tests: **a** and **b**—Test 1; **c** and **d**—Test 2; **e** and **f**—Test 3; **g** and **h**—Test 4; **i** and **j**—Test 5; **k** and **l**Test 6; **m** and **n**—Test 7; **o** and **p**—Test 8; **q** and **r**—Test 9; **s** and **t**—Test 10; **u** and **v**—Test 11; **w** and **x**—Test 13; and **y** and **z**—Test 13

The fitting errors are plotted in Figure 16 in the Appendices in the form of bar graphs along both the axial and circumferential directions. With few exceptions, the error bar graphs reveal that a large portion of the fitting errors exist in large tissue stretches ($\lambda >1.4$). Most of the toe region errors occur along the circumferential direction. Tests #3, #5, and #7, however, exhibit large errors in both directions within the toe region. In all these cases, it is observed that the esophageal tissues were too compliant, as a result of which the stress curves had very low tangential moduli. It is not immediately clear what might have caused such high compliance, although it could be attributed to tissue damage at the supports and the effects of lack of preconditioning. The tangential moduli of Tests #3, #5 and #7 were much smaller than the c parameter in the Holzapfel model, which were 9.1 kPa and 5.1 kPa in the axial and circumferential directions, respectively.

## Discussion

Phenomenological modeling is the leading modeling approach for esophageal or gastrointestinal tissue [21], accounting for up to 56% of all models, with Fung-type accounting for 35%. Besides some limitations to the current modeling approaches, such as limited availability of human esophagus data, limited viscohyperelasticity studies, difficulties relating to the validation of electromechanical models for estimation of active behavior, and complexity of microstructural models for structure–function prediction, there is generally a deficiency in the current deterministic models in that they fail to capture the widely diverse behavior of the esophageal tissue subjected to different operating conditions. Most of the current models for prediction of passive behavior of esophageal tissue are based on exponential strain energy function [49]. However, questions relating to model fidelity and ability to predict a rather random tissue behavior remain. The mechanical behavior and performance of the soft tissues is critical in developing therapies for various diseases [50–53], and these soft tissue mechanics have been utilized in further understanding of disease mechanisms [52, 53].

In the study, the tensile behavior of the esophageal tissue in axial and circumferential directions was assessed. The Holzapfel and NARX models were used to predict the tissue behavior in these directions. The results show that both models have very high correlations, which means that they are able to capture the essential tissue behavior of nonlinear elasticity. The only difference occurs in the mean square error, where the NARX model yields better fit than the Holzapfel model. The major contribution to the errors is from large tissue stretches (stretch ratios greater than 1.4), although there are quite a few cases where the errors occurred in regions of small tissue stretches, probably due to esophageal tissue damage at the supports and lack of tissue preconditioning. For the training of the NARX network, three different forms of training data were explored: raw test data, linear curve-fitted data, and Holzapfel model-fitted data. Although the NARX model showed slightly better performance when trained with the Holzapfel model-fitted data, the difference in performance arising from the use of these three different types of training data was not statistically significant. The only significant advantage of using Holzapfel model-fitted data, therefore, was that the results could easily be interpreted to extract important material properties of the esophagus based on its initial values in the training data.

In the study, inadequate data preprocessing and incorrect selection of training data were found to be two of the most important sources of network error. Inadequate preprocessing of the training data was found to yield a neural network with insufficient features to simulate accurately on some of the test data. This is a huge concern in the case of tests such as these, where the behavior of the tissue is not exactly repetitive, and the stress curves are often widely separated from one another at large stretch ratios. For esophageal tissue, it is reported that such mechanical differences are region dependent, which implies that there can be differences in esophageal tissue harvested from different regions of the same organ [6]. This difficulty was treated in the study by applying a data normalization procedure, where all the training data were scaled according to the maximum tensile stresses. A failure to apply such normalization resulted in saturation errors during network simulation. The selection of training data was key in identifying and extracting physically meaningful physical features of the studied esophageal tissue. In the study it was found that not all test data could produce such physically meaningful features. The parameter that was monitored in the study was the tangential modulus. A candidate for inclusion as training data needed to have a positive value of the c material parameter in the case of the Holzapfel model-fitted data or a positive slope in the case of the linear curve-fitted data. Negative slopes resulted in forcing the neural network outputs in the opposite direction when simulated on some of the tests.

Two other significance tests were conducted on the effects of changing levels of the added noise and effects of combining different training data. It was found that the change in the level of the added noise did not yield any statistically significant differences in the accuracy of the network simulation, so the noise level was maintained at a magnitude of 1 kPa. However, a combination of poor and good training data produced statistically significant results as expected. The strength of the differences depended on their relative influence in the combined training data. For example, combined training data in which the poor training data had more influence than the good training data would show differences that were statistically significant in the direction of the poor data and vice versa.

In terms of the validity of the tensile test results obtained in the study, axial tensile strength of the esophageal tissue was found to be much higher than that along the circumferential direction; this bears out the work of other researchers [6, 7, 22]. In some cases, the axial stresses were found to be approximately twice as great as those in the circumferential direction, which is in agreement with the relationship between stresses in the axial direction and circumferential direction [55] in perfectly thin cylinders. However, this was not true in all cases in the study reported on here, probably due to the fact that the esophagus is not a perfect cylinder and inflation tests were not conducted in this case.

The stress results in this test were, however, below those reported by other researchers working on sheep models [22]. These differences may be due to the differences in the gender, species, age and testing conditions (specifically lack of preconditioning may have played a major role here). It is also possible that the differences in tissue preparation might have contributed towards such differences, since it is noted that in some of the studies by other researchers, the specimens were separated into different layers of the esophagus [6, 22, 54]. For example, [22] tested only the mucosa–submucosa layer, while [6] compared the tensile strength of the mucosa–submucosa with the muscle tissue of the esophagus. In this study, the gloss anatomy of the esophagus was tested biaxially, and the effects of the softer adipose and muscle tissue may have affected the overall tensile strength of the tested specimens.

## Conclusions

In this paper, the NARX network was applied to the modeling of tensile behavior of ovine esophagus. The tissue was stretched in axial and circumferential directions using the CellScale biaxial tester. A total of 13 tests were recorded. Average stress data were calculated and plotted over all 13 tests as stress–strain curves. It was found that the test data had wide standard deviations with regard to the average stress curve between 77 and 166% for axial stresses and between 71 and 154% for circumferential stresses. The largest contribution to these deviations was from the large stretch ratios. These differences in the measured data made it extremely hard to predict stress responses on the other tests using material parameters that were obtained on any single measured data. For example, it was found that material parameters obtained by fitting the Holzapfel model to the average stress data yielded fitting errors in excess of 200% when applied to some of the tests. Therefore, stress response prediction using constitutive models may be extremely difficult.

As a result, the capabilities of an artificial neural network were investigated. A 1-5-1 NARX network was identified, and it was found to yield fitting errors within 16% at a 99.9% correlation level. The network was trained using data that were obtained by fitting a Holzapfel model to Test #6. With this, it was then possible to infer nominal values of material parameters for the esophageal tissue derived from the Holzapfel model. For the sheep esophageal tissue under study, it was found that fibers were oriented at angles of 57.3° and 91° from the circumferential axis. This showed them to be more aligned towards the axial direction than the circumferential direction, which might explain the relatively greater stiffness of the tissues along the axial direction.

There are, however, two key observations that influenced the accuracy of the trained neural network:

(i) Training data had to be properly scaled and normalized to avoid saturation when the network was trained on tests whose values lay far from the training data.
(ii) The selection of the training data had to be done in such a way that the slopes of the stress–strain curves in small stretch ratios were sufficiently high and positive. In the study, the c parameter for the Holzapfel model had to be positive to avoid driving the network weights in the opposite direction.

## Materials and methods

### Specimen preparation

Esophageal tracts harvested from 13 Vleis merino sheep weighing between 40 and 42 kg were collected from a local abattoir [55]. The organs were transported in cooler boxes packed with ice to the University of South Africa’s Biomedical Engineering test laboratory within 4 hours. The esophageal tracts measured between 40 and 50 cm long. The specimens used in this study were dissected around the medial sections of the tracts at 10 mm away from either side of the midline. Thus, there were 13 20-mm-long test samples that were initially cut as shown in Fig. 12(a). The tubular samples were later cut open to yield 20 mm by 40 mm specimens as shown in Fig. 12(b). The 13 specimens were then carefully washed of all food items in water at room temperature without necessarily removing the excess adipose tissue. The direction in which food passes was marked as the longitudinal/axial direction, while the perpendicular direction was marked as the circumferential direction [24]. All samples were preserved in saline buffer solution at room temperature (approx. 22 °C) to maintain freshness until testing.

### Equipment set-up and testing

A CellScale biaxial testing machine [55–58] was used to test the specimens. The machine was set up for displacement control at strain rates fluctuating between 8%/s and 4%/s as shown in Fig. 13**.** As shown in the figure, the strain rates decreased from slightly above 8%/s to 4%/s with increased stretches. Although the equipment was set for displacement control, the effects of tissue stiffening appeared still to be evident in the results. The machine was set to stretch the tissue equally in both directions up to a strain level of 50% for 8.5 s. The temperature of the batch was set to 38  C to match the body temperature of the sheep [55]. A preload of 0.5 N was applied for each test to remove any existing slackness in the specimens. No preconditioning cycles were applied to the test samples. Lack of tissue preconditioning may have neglected the effects of fiber realignment and molecular reorganization that could have occurred during the period of transportation, tissue preparation and storage prior to testing.

Bio-rakes were used to hold the specimens during tensile testing. The use of bio-rakes ensures equal distribution of the pulling forces [59] on the test specimen, and it also ensures that the slip of the tissue at the clamping sites is substantially reduced. Between any two successive testing cycles, there was a rest time of approximately 10 min. The machine was recalibrated before each test was carried out. The testing of the specimens lasted for an extra 3 h after arrival at the laboratory. However, due to strict preservation procedure, they were assumed to be fresh samples. Sommer et al. [60] ovine esophageal tissue collected from an abattoir were cleaned of all fat and cud. The tests were completed in under 36 h, and tested at 4 °C in phosphate-buffered solution (PBS) to avoid proteolysis and tissue property deterioration. In a study by Perlink et al. [61], the interaction between stent and ovine esophagus was investigated at a temperature of 37 °C in PBS for a total duration of 10 weeks. Comparatively, the total duration for the test in the current study was much shorter and completed within a very short time from tissue harvesting.

## Data Availability

The research data can only be made available on request from the corresponding author.

## Appendix 1

Appendix A contains more graphs of the results for the training and simulation performance of the artificial neural networks. These graphs could not all be accommodated within the paper’s main text. Figure 14 presents the regression performance of the NARX network for the three formats of training data after being randomly divided into three parts as 50% for training, 25% for testing and 25% for validation labeled as “Training”, “Test” and “All”, respectively, in the graphs. Figure 15 presents the NARX predictions for all 13 tests in both axial and circumferential directions plotted over the experimentally acquired stress–stretch ratio data. The histograms in Figure 16 show the errors between the ANN-predicted stresses and the measured ones.
